# Nuclear Dynamics in Quiescent Cells: Conserved Mechanisms from Yeasts to Mammals

**DOI:** 10.3390/biom16020203

**Published:** 2026-01-28

**Authors:** Sigurd Braun, Cornelia Kilchert, Aydan Bulut-Karslioglu, Myriam Ruault, Angela Taddei, Fatemeh Rabbani, Dominika Włoch-Salamon

**Affiliations:** 1 Institute of Genetics, Justus Liebig University Giessen, 35392 Giessen, Germany; 2 Institute of Biochemistry, Justus Liebig University Giessen, 35392 Giessen, Germany; 3 Max Planck Institute for Molecular Genetics, 14195 Berlin, Germany; 4 UMR 3664 Nuclear Dynamics, CNRS, Institut Curie, Université PSL, Sorbonne University, 75248 Paris, France; 5 Institute of Environmental Sciences, Faculty of Biology, Jagiellonian University, 30-387 Kraków, Poland

**Keywords:** cellular quiescence, nuclear organization, chromatin remodeling, transcriptional reprogramming, post-transcriptional regulation, epigenetic regulation

## Abstract

Quiescence is a reversible, non-proliferative cellular state that enables survival under nutrient limitation while preserving the capacity to resume growth. Rather than representing a passive default, quiescence is an actively regulated program conserved from unicellular eukaryotes to metazoans. This review focuses on the nuclear mechanisms underlying quiescence entry, maintenance, and exit, with primary emphasis on mechanistic insights from yeast models while highlighting conserved principles in multicellular systems. Across species, quiescence is characterized by global transcriptional repression, chromatin compaction, and the extensive reorganization of nuclear architecture, coordinated by nutrient-sensing pathways centered on TOR/mTOR signaling. We discuss how transcriptional reprogramming is achieved through redistribution of RNA polymerases, dynamic transcription factor activities, and large-scale remodeling of histone modifications, alongside repressive chromatin formation. In parallel, post-transcriptional mechanisms—including intron retention, alternative polyadenylation, and accumulation of non-coding RNAs—fine-tune gene expression while limiting biosynthetic output. We further examine how changes in nuclear organization, such as nucleolar condensation, condensin-mediated chromosome rearrangements, and telomere hyperclusters, support long-term viability and genome stability. Collectively, this review highlights nuclear dynamics as an integrative regulatory layer that links metabolic state to cellular identity, adaptability, and long-term survival, with broad implications for development, stem cell function, and disease.

## 1. Introduction

**Figure 1:**
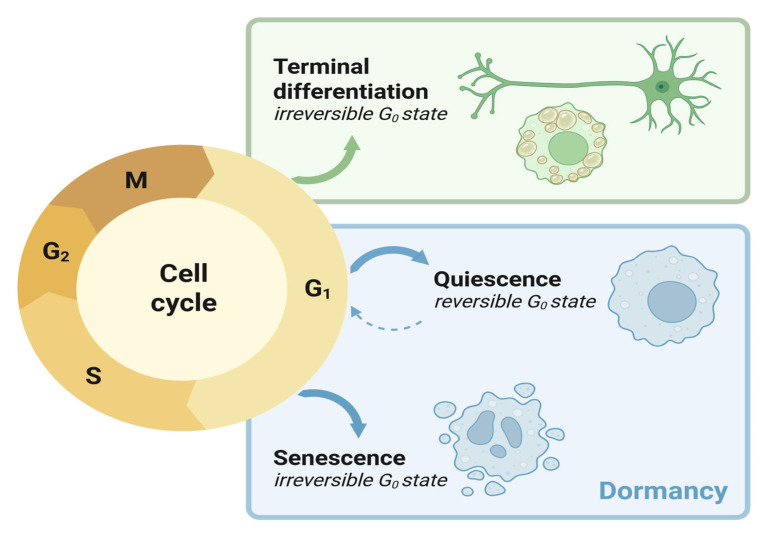
**Definitions of different non-proliferative cellular states.** Non-proliferative cells can exist in several distinct states that differ in their reversibility and developmental context. However, terminology varies considerably across fields: the term “dormancy,” for instance, has been applied inconsistently to describe reversibly arrested cells, irreversibly arrested cells, or both, depending on the system and context. To avoid ambiguity, we primarily use the more stringently defined terms “quiescence” and “senescence” throughout this review. Quiescence refers to a reversible cell cycle arrest in which cells retain the capacity to resume division upon appropriate stimulation. Examples include starved cells, adult stem cells, immunological memory cells, dormant cancer cells (often termed cancer stem-like cells or persister cells), and diapaused embryos. Senescence is characterized as an irreversible cell cycle arrest that can arise as a consequence of aging, DNA damage, or other forms of cellular stress. Terminal differentiation, in contrast, describes cells that exit the cell cycle as part of a developmental program, acquiring specialized functions that typically preclude further division. Created in BioRender. Kilchert, C. (2026) https://BioRender.com/85t3r35 (accessed on 16 January 2026).

Quiescence, the reversible arrest of cell proliferation, is the predominant state of life. Unlike terminally differentiated and senescent cells, quiescent cells (Q cells) maintain the full capacity to return from G0 and reenter the cell cycle (Figure 1). Unicellular organisms benefit from quiescence by surviving periods of growth limitation and resume active proliferation when favorable conditions return. Quiescence is also essential for various aspects of metazoan biology including reproduction, immunological memory, and tissue regeneration [1,2]. For example, unfertilized mammalian oocytes are generated during fetal development but can remain dormant for decades until release during ovulation. Even after fertilization, embryonic development can be temporally suspended: in some organisms, diapause, a form of developmental dormancy, is triggered to increase the chances of embryo survival under starvation or other stressors. In multicellular organisms, quiescent tissue-resident stem cells serve as reservoirs for tissue homeostasis and regeneration. At the same time, Q cells in tumors and fungal biofilms—often termed persister cells—also contribute to therapy resistance and disease recurrence, underscoring the clinical relevance of understanding quiescent cell biology.

**Figure 2:**
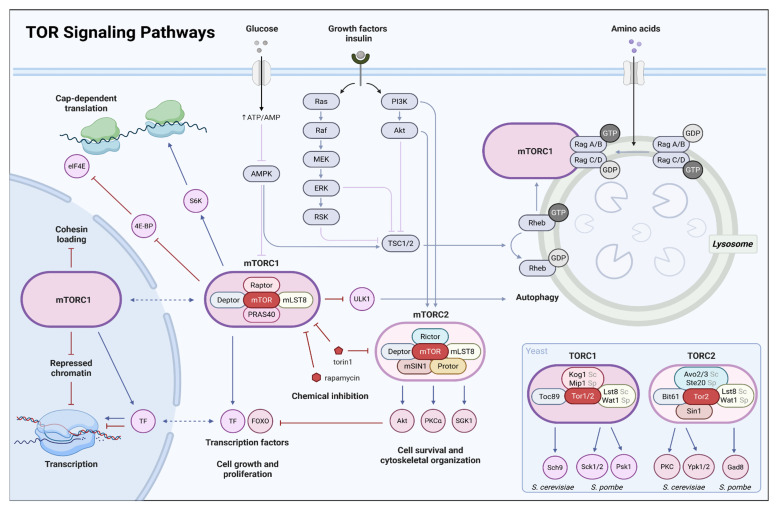
**mTOR signaling integrates information on nutrient availability to regulate cell growth and proliferation.** Signaling from different nutrient-sensing pathways converges on the mechanistic target of rapamycin (mTOR) kinase, which assembles into two different complexes, mTORC1 and mTORC2, to regulate central cellular processes related to growth and survival. In the presence of amino acids, guanine nucleotide exchange enables the Rag A/B-C/D complex to recruit mTORC1 to the lysosomal surface, where it can be activated by the small GTPase Rheb in its GTP-bound state. High Rheb-GTP levels are maintained in response to growth factor and insulin signaling via protein kinase cascades (including the kinases AKT and ERK) that inhibit the tuberous sclerosis complex (TSC1/2), a GTPase activating protein for Rheb. Activated mTORC1 phosphorylates cytoplasmic substrates and regulates nuclear transcriptional programs to promote growth. Key targets include Unc-51 Like Autophagy Activating Kinase 1 (ULK1; inhibitory phosphorylation suppresses autophagy); eIF4E-binding proteins (4E-BPs; phosphorylation prevents inhibition of cap-dependent translation); and S6 kinase (S6K), which phosphorylates diverse substrates including ribosomal protein S6 and transcription factors. In the nucleus, mTORC1 signaling maintains chromatin in an active state permissive for transcription. mTORC1 signaling is further modulated by cellular energy status via AMP-activated protein kinase (AMPK), which is activated when ATP levels drop. Growth factor signaling through phosphoinositide-3 kinase (PI3K) and AKT also activates mTORC2 at the plasma membrane, which regulates cell survival and cytoskeletal organisation. mTORC2-dependent phosphorylation retains the key transcription factor FOXO in the cytoplasm, and its regulation of acetyl-CoA pools modulates histone acetylation levels. Inhibition of mTOR signaling—either by nutrient deprivation or chemical inhibitors such as rapamycin or Torin1—triggers entry into quiescence. Created in BioRender. Braun, S. (2026) https://BioRender.com/ki389l6 (accessed on 17 January 2026).

Quiescence is believed to be an early evolutionary phenomenon that is universal across life. Periods of hardship and nutrient scarcity are inevitable companions of life—when conditions are favorable, life explodes, and intense competition for nutrients, and their depletion, are the consequence. It begs no question what an immense advantage the ability to withstand poor conditions must have posed from life’s inception. In the course of evolution, quiescence has been non-uniformly adapted to suit the specialized purpose of diverse biological contexts [1]. However, despite the diversity and complexity of eukaryotic cellular arrest models, cellular processes associated with entry into, maintenance, and exit from the quiescent state are often shared. Across eukaryotes, quiescence is characterized by reduced cell size, chromatin compaction, and global downregulation of transcription and translation, reflecting a profound metabolic shift in which cells suppress growth-associated biosynthesis while selectively activating catabolic pathways and antioxidant defenses that support long-term survival [3]. Across organisms, these changes are driven by starvation-responsive signaling pathways, prominently including inactivation of the highly conserved Target of Rapamycin (TOR) kinase complexes or its metazoan homolog Mechanistic Target of Rapamycin mTOR (for key terms and abbreviations, see Table 1). TOR and mTOR integrate information from multiple nutrient-sensing pathways and promote cell growth, proliferation, and survival when conditions are favorable; their inactivation initiates a conserved “hold and protect” program that gradually develops into quiescence [4,5] (Figure 2). The central nature of TOR signaling in quiescence establishment is underscored by the fact that pharmacological TOR inhibition, for example with rapamycin, recapitulates most hallmarks of quiescence even in complex systems, and has been used experimentally as a model of diapause. For example, partial inhibition of mTOR in mouse blastocysts and embryonic stem cells induces a reversible paused state that preserves pluripotency, reduces transcription and translation, alters histone modifications such as H4K16ac and H3K36me2, and allows full developmental reactivation upon release from inhibition [6]. Importantly, TOR signaling is not restricted to its well-characterized cytoplasmic functions in translational control and metabolism. In addition to regulating the nucleocytoplasmic distribution of downstream signaling effectors, both TORC1 and TORC2 possess active nuclear pools that shuttle between the cytoplasm and nucleus and associate directly with chromatin, positioning TOR as a direct regulator of gene expression and epigenetic state [5] (Figure 2).

Together, these observations establish quiescence as a highly regulated, multilayered program rather than a uniform arrested state. By integrating cytoplasmic control of metabolism and translation with direct nuclear regulation of transcription, chromatin organization, RNA processing, and epigenetic stability, adaptations during quiescence enable cells to survive prolonged periods of unfavorable conditions while retaining the capacity for rapid reactivation when environments improve. This kind of rewiring is found in unicellular organisms as well as metazoan models, but can exhibit substantial heterogeneity across cell types and even among clonal cell populations [3,7,8,9,10,11,12,13]. This apparent contradiction between universal molecular pathways and diverse cellular responses raises critical questions: How can conserved molecular mechanisms give rise to such distinct phenotypic outcomes? And what cell type-specific factors shape quiescence dynamics?

**Genetic screens have revealed the central importance of nuclear processes.** Screens using yeast deletion collections or temperature-sensitive mutants have confirmed the central importance of TOR signaling, repression of protein biosynthesis, and an increase in autophagy in establishing quiescence [3], while also identifying numerous genes that are essential for survival during G0 or for re-entry into the cell cycle [14,15]. Notably, genes governing nuclear dynamics were frequently recovered in these screens, reflecting the extensive remodeling of nuclear architecture and chromatin reorganization that accompanies entry into quiescence across organisms [16]. This observation prompted Zahedi et al. to perform a targeted flow cytometry screen of known chromatin regulator deletion mutants in fission yeast [17], monitoring the proportion of cells in G0 as well as cell mortality over extended periods of quiescence to distinguish between genes essential for entering G0 and those required for long-term viability. Strikingly, among genes annotated with the Gene Ontology term “chromatin organization”, 68% of the mutants had defects in establishing or stably maintaining quiescence. Similarly high defect rates were observed for genes involved in “chromatin remodeling” (69%), “regulation of transcription” (71%), or “DNA repair” (76%), underscoring the importance of nuclear processes during cellular quiescence [17,18].

**Scope and organization of this review.** Despite the accumulating evidence for the critical role of nuclear remodeling during quiescence, our understanding of the underlying mechanisms remains incomplete. Here, we provide a comprehensive overview of current knowledge regarding nuclear dynamics during quiescence entry, maintenance, and exit, with an emphasis on features conserved across eukaryotes (Table 2). Because most mechanistic data derive from yeast models, we focus primarily on these systems, while highlighting similarities and differences in higher eukaryotes where data are available. Throughout, we emphasize TOR signaling as a master coordinator of nuclear remodeling: strikingly, many complex nuclear rearrangements in Q cells can be triggered by TOR inhibition alone, suggesting TOR translates quiescence-inducing signals into specific architectural and functional adaptations of the nucleus. We link observed changes in nuclear dynamics to their upstream regulation by TOR signaling where such mechanisms are understood, while also pointing out knowledge gaps. For readers interested in the mechanistic details of TOR function and signaling in the nucleus, we refer to a comprehensive review [5]. Topics beyond our scope, including genome stability and DNA damage repair, as well as non-nuclear aspects of quiescence, including signaling pathways, metabolic reprogramming, and the temporal dynamics of quiescence regulation, are covered in recent publications and dedicated reviews [2,8,19,20,21,22].

## 2. Established Models for Studying Nuclear Dynamics During Quiescence

### 2.1. Unicellular Eukaryotic Models

Yeast species are powerful models for studying both cellular proliferation and quiescence. *Saccharomyces cerevisiae* (budding yeast) and *Schizosaccharomyces pombe* (fission yeast) have been particularly instrumental in identifying essential conserved mechanisms involved in quiescence establishment. These species diverged 300–350 million years ago, yet they share fundamental cellular processes. Mechanisms conserved between these two evolutionarily distant yeasts are therefore likely to be relevant for metazoans as well. In both organisms, nutrient deprivation leads to a reversible cell cycle arrest and the transition into a quiescent state.

**Budding yeast (*Saccharomyces cerevisiae*).** *S. cerevisiae* has served as a leading eukaryotic model for quiescence research [19]. Early studies showed that cultures starved for carbon, nitrogen, or phosphate accumulate small G1-arrested cells [23], which were later referred to as G0 cells [24,25,26,27]. An extensive body of work has led to the development of established guidelines, toolkits and databases that facilitate experimental design and analysis. However, variation in strain background, chronological age, starvation protocols, and criteria to identify Q cells in yeast has resulted in at least 48 possible experimental setups across published studies [28]. One commonly used approach induces quiescence through exhaustion of rich medium, resulting in gradual depletion of the carbon source. Exhaustion or abrupt starvation of other key nutrients (nitrogen, phosphate) also triggers a quiescent state, for which the specific cellular responses can differ. Importantly, the path used to trigger quiescence leads to very different functional outcomes, including gene expression profile, stress resistance, and lifespan [2]. For instance, exponentially growing cells abruptly starved from glucose will rapidly die, while cells from a stationary phase culture will survive in water for several weeks [29]. This is why Q cells resulting from the progressive exhaustion of rich medium are commonly used to study chronological aging [4]. It also emphasizes the importance of providing precise descriptions of experimental methods.

Work from the Werner-Washburne laboratory demonstrated that cells in stationary-phase cultures are heterogeneous and can be separated based on density. Most properties attributed to deep stationary-phase cells are, in fact, features of the dense fraction. This dense population corresponds to Q cells (or deep Q cells), whereas the less dense fraction is referred to as NQ cells (for non-Q). NQ cells more rapidly transition into a senescent state and lose the ability to reenter the cell cycle during prolonged dormancy. Q cells begin to differentiate as glucose levels decline [30,31], prior to the metabolic transition from fermentation to respiration known as the diauxic shift (DS). They are predominantly small G1-arrested daughter cells, whereas NQ cells are more heterogeneous in both size and cell-cycle phase. Q cells are also characterized by a thickened cell wall that enhances thermotolerance [29]. Notably, the relative abundance of Q and NQ cells varies across genetic backgrounds, contributing to discrepancies in the literature. Strains with respiration defects, such as the commonly used BY background, produce lower yields of Q cells than W303 strains [32]. Auxotrophies—disruptions in key metabolic pathways introduced to facilitate genetic manipulation—also limit the ability of cells to enter the Q-cell state. It is therefore recommended to minimize auxotrophies and to culture cells under conditions that favor respiration [2].

**Fission yeast (*Schizosaccharomyces pombe*)**. *S. pombe* is predominantly haploid and contains three chromosomes, with a genome size comparable to that of *S. cerevisiae*. Although these two yeast diverged early and differ substantially in genome organization, chromosome architecture, and regulatory pathways, they retain many conserved cellular processes; nonetheless, *S. pombe* offers complementary advantages as a quiescence model [16]. Quiescence in *S. pombe* is most commonly induced by nitrogen starvation. Starved cells undergo two more rounds of cell division without cellular growth and then arrest in G1, facing two possible outcomes: in the presence of opposite mating types, cells initiate a mating program that ultimately produces resilient spores. In contrast, heterothallic strains—which are fixed in one mating type and self-infertile—progressively enter the G0 state, in which they can remain viable for weeks or months in the presence of a carbon source [33]. Alternative conditions, such as glucose elimination or growth to saturation, cause G2 and G1 arrest, respectively, which resemble nitrogen starvation-induced quiescence but fail to support long-term cell survival [16]. Unlike *S. cerevisiae*, *S. pombe* does not have enzymes for the glyoxylate cycle required for ethanol metabolism and therefore does not undergo the DS. An important experimental advantage of nitrogen starvation is that G0 entry and exit occur synchronously within the cell population [16,34].

### 2.2. Multicellular Models

Temporary entry into quiescent states in response to developmental or environmental cues is widespread in multicellular organisms, which are composed of complex tissues containing diverse cell types that must function in coordination. Quiescence programs can be executed by specific lineages, such as neuroblasts in *Drosophila* or the germline in *C. elegans.* Alternatively, quiescence can affect the entire organism, as exemplified by the *C. elegans* dauer diapause. These forms of quiescence have been extensively studied and are the subject of several recent reviews [35,36,37].

**Adult stem cells.** Many multicellular tissues harbor stem or progenitor cells that sustain tissue integrity under homeostatic conditions and, when required, mediate repair and regeneration [38]. In most cases, a subset of these cells remains in a quiescent state and becomes activated only in response to defined physiological cues. These tissue-resident adult stem cells occupy specialized niches—microenvironments composed of neighboring cells, extracellular matrix components, and soluble factors—that protect them from cellular damage by restricting replication-associated and metabolic activity. Adult stem cells therefore constitute highly informative models for studying quiescence. They share characteristic features, including low RNA content and absence of proliferation markers, and frequently coexist with more rapidly cycling progenitor pools within the same tissue compartment. Their behavior is tightly regulated by niche-derived signals, and disruption of these cues, for example during ageing, results in impaired stem cell activation and tissue decline. Across systems such as muscle stem cells (MuSC), hematopoietic stem cells (HSC), hair follicle cells (HFSC), and neural stem cells (NSC), quiescence is associated with a distinct epigenetic landscape. Among these, HSCs represent a paradigmatic example of adult stem cell quiescence. In adult mammals, HSCs reside in specialized bone-marrow niches, where a subset of long-term HSCs remains deeply quiescent for extended periods, dividing only rarely [39,40]. Lineage-tracing and label-retention studies indicate that HSCs contribute little to steady-state hematopoiesis, which is instead largely sustained by more actively cycling progenitors. However, in response to stresses such as inflammation or irradiation, HSCs reversibly re-enter the cell cycle and regenerate the hematopoietic system, underscoring quiescence as a protective yet readily deployable state [41,42,43]. This functional separation between quiescent stem cells and active progenitors mirrors principles observed across adult stem cell systems, reinforcing their value as general models for quiescence. For a detailed overview of the regulation of adult stem cell quiescence, the reader is referred to a recent comprehensive review [21].

**Dormant mouse embryo**. Systemic quiescence in mammals is rare, occurring only in unfertilized oocytes and during early embryonic development. While adult stem cells maintain quiescence to support tissue homeostasis, repair, and regeneration, embryos employ dormancy as a reproductive strategy—either to optimize the timing of birth or to survive environmental challenges—a phenomenon known as embryonic diapause [44]. In the context of embryonic diapause, the period of dormancy can last from days to months, depending on the species [45]. In most cases, the embryo pauses at the blastocyst stage, delaying implantation, and further development. Documented to date in over 130 mammalian species, diapause remains a mystery due to the inaccessibility of embryos from most wildlife species. Mouse models have been instrumental in studying diapause, as it can be reliably induced through ovariectomy or hormone treatment [46,47]. Over the last decade, in vitro models of diapause have been developed and, together with advances in detection methods, have significantly propelled the field forward.

Similar to unicellular organisms, the central cellular growth regulator mTOR is a key factor controlling diapause in mammals [6,48]. Additionally, nutrient depletion, suppression of insulin signaling, Myc activity, and lipid metabolism have been documented to trigger a diapause-like response [6,49,50]. For a detailed comparative analysis of dormancy in adult stem cells and embryos, readers are referred to [51].

While quiescence is used across various tissues in multicellular organisms to enable preservation and repair, for the remainder of the review, we will mainly focus on its use and regulation in the early mammalian embryo. The undifferentiated progenitor cells within the early embryo are in many ways reminiscent of yeast, exhibiting low heterochromatin load and globally high transcriptional activity, and employing similar strategies to adapt to their immediate environment.

## 3. What Drives Transcriptional Reprogramming in Quiescent Cells?

### 3.1. Transcriptional Reprogramming During Metabolic Transitions in Yeast

In log cells, TORC1 directly regulates transcription by all three RNA polymerases (RNAPI, II, and III). It binds promoters of rDNA and tRNA genes to stimulate transcription, while modulating RNAPII-dependent expression of metabolic genes through interactions with transcription factors and coactivators. Moreover, TORC1 promotes transcriptionally permissive states by stimulating histone acetylation, antagonizing histone deacetylases including sirtuins, influencing histone methylation, and coordinating ATP-dependent chromatin remodeling complexes that maintain open promoter architecture [5]. As cells transition into quiescence, cellular processes shift from biomass production to maintenance. This is reflected in profound changes to the transcriptome and, to a lesser extent, the proteome. Global transcriptional repression results in a marked decrease in growth-related mRNAs, for example those encoding ribosomal proteins, while the relative expression of genes involved in nutrient uptake and scavenging increases. In *S. cerevisiae*, glucose deprivation results in an approximately 30-fold reduction in mean transcript abundance [52,53,54]. Similarly, measurements of absolute molecule numbers in fission yeast have revealed a global reduction in RNA levels upon nitrogen starvation, even when accounting for the smaller size of Q cells [55]. Recent studies have identified several mechanisms that likely act in concert to robustly limit mRNA production under these conditions.

In *S. cerevisiae*, quiescence is established over the course of several days, with Q cells exhibiting distinct transcriptional reprogramming. RNAPII distribution undergoes significant changes, characterized by reduced levels of both initiating and elongating forms of RNAPII [53]. Different studies have reported distinct RNAPII distributions, likely reflecting differences in the kinetics of quiescence entry arising from variations in growth conditions, such as aeration and medium pH [28,56]. Monitoring RNAPII over time revealed the evolution of RNAPII distribution during entry into quiescence [57]. Shortly after the DS (24 h of culture), RNAPII decreases in promoter-proximal regions and accumulates near transcription end sites (TES), potentially reflecting regulatory changes in transcription termination [53]. RNAPII then accumulates slowly at upstream-activating sequences of one third of coding genes in late Q cells [57,58,59]; intergenic enrichment correlated with fast resumption of transcription upon quiescence exit. As mentioned above, this decrease in RNAPII levels on chromatin accompanies a global transcription shut-down [52,53,54]. Nevertheless, the majority of transcripts remain detectable in mature Q cells, with 5105 genes expressed compared to 6205 in logarithmically growing (log) cells [53]. Notably, approximately 40% of these RNAs are found in an extraction-resistant compartment that is sensitive to protease treatment, indicative of sequestration in RNA-protein (RNP) complexes [53,60]. Consistent with this, two types of RNP granules are observed in Q-cells: processing bodies (P-bodies) and stress granules. While the former assemble after the DS upon PKA inactivation, the latter do not assemble until cells have reached the stationary phase [61]. Although most genes are shut down, approximately 3% of coding genes remain active, with seven detected in more than 80% of the cells in single-cell transcript analyses [57], indicating a very high-level of expression.

Similarly, *S. pombe* exhibits pronounced global repression of transcription during quiescence, with total mRNA levels reduced to approximately 15% of those in log cells [55]. This is accompanied by decreased levels of RNAPII engaged in transcription initiation and elongation [62]. Transcriptional reprogramming following nitrogen deprivation is dynamic, and distinct profiles are observed during the initial response (1–2 h) and the establishment phase (10–24 h) [63]. During establishment, several hundred genes, many of them involved in stress responses, detoxification, and recycling of amino acids and nucleotides, are transiently activated [55,63,64]. Once stable quiescence is achieved, expression of these transiently induced genes is decreased, and 97% of the genome becomes downregulated. Only 16 ‘core quiescence genes’ remain highly expressed even after two weeks of nitrogen starvation [64].

### 3.2. Transcription Factor Dynamics Orchestrate Chromatin Remodeling

What then drives the extensive transcriptional reprogramming during quiescence entry? In *S. cerevisiae*, differential gene expression is coordinated by a network of transcriptional activators and repressors. Their activity is tightly regulated by integrated signaling networks that include PKA, TORC1, Snf1, and Pho85, which collectively regulate metabolism, stress resistance, translation, autophagy, and the accumulation of energy reserves, such as glycogen, trehalose, and neutral lipids [2,3].

**Global gene regulation through altered activities of transcriptional activators and repressors.** In the presence of glucose, the C2H2-ZF transcription factors Mig1, Mig2, and Mig3 repress hundreds of genes. When glucose is depleted, Mig1 and Mig2 relocalize to the cytoplasm upon phosphorylation by the yeast AMPK Snf1, while Mig3 (and also Mig2) become transcriptionally repressed [2,65,66]. Upon the DS, the C2H2-family transcription factors Msn2 and Msn4 induce expression of a broad set of glucose-repressed genes through binding to specific stress-response elements (STREs) present in their promoter regions [2,67]. Msn2 also contributes to large-scale chromatin reorganization by promoting condensin recruitment to STRE-containing genes, thereby regulating higher-order chromatin structure (see Section 5.2). Msn2/4 activity is negatively regulated by the TORC1 and PKA pathways and positively regulated by the Rim15 protein kinase, which acts downstream of TORC1, through differential phosphorylation that controls nuclear localization of Msn2 and Msn4 [2,68].

Another layer of regulation is imposed by two transcriptional repressors, Xbp1 and Stb3. Xbp1 is transcriptionally induced by Msn2/4 during the DS and other forms of stress and becomes one of the most abundant transcripts in Q cells [2,31,69]. Its accumulation correlates with the frequency and duration of stress, suggesting that Xbp1 serves as a “stress memory” [2]. Xbp1 binds to specific promoter motifs present in over 500 genes, including the G1 cyclin *CLN3* [2,31]. Stb3, in contrast, binds ribosomal RNA processing elements (RRPEs) and represses ribosomal biogenesis (RiBi) gene expression. Its recruitment to ribosomal protein gene (RPG) promoters is inhibited by TOR-dependent phosphorylation, which prevents Stb3 nuclear localization [70,71]. Both repressors are intimately linked to dynamic changes in histone modifications.

**Altered histone modifications and nucleosome density.** Histone acetylation is typically associated with active transcription by modulating nucleosome structure and promoting the recruitment of effector proteins or ‘readers’. In addition, lysine methylation, occurring both at N-terminal tails and within core histone domains, is associated with transcriptional activation in budding yeast. Transcriptional repression in Q cells is accompanied by a global increase in histone density and a marked decrease in acetylation at multiple residues at histone H3 and H4 [2,52,54,72]. These chromatin changes are initiated early during the DS, particularly at ribosomal genes, but become fully established after six days in stationary culture. Although histone hypoacetylation occurs genome-wide, gene-specific patterns emerge: growth-related genes exhibit a pronounced decrease in acetylation, whereas stress-response genes upregulated in quiescence gain acetylation marks, indicating a tight correlation between histone acetylation and transcriptional activity [52].

**Figure 3:**
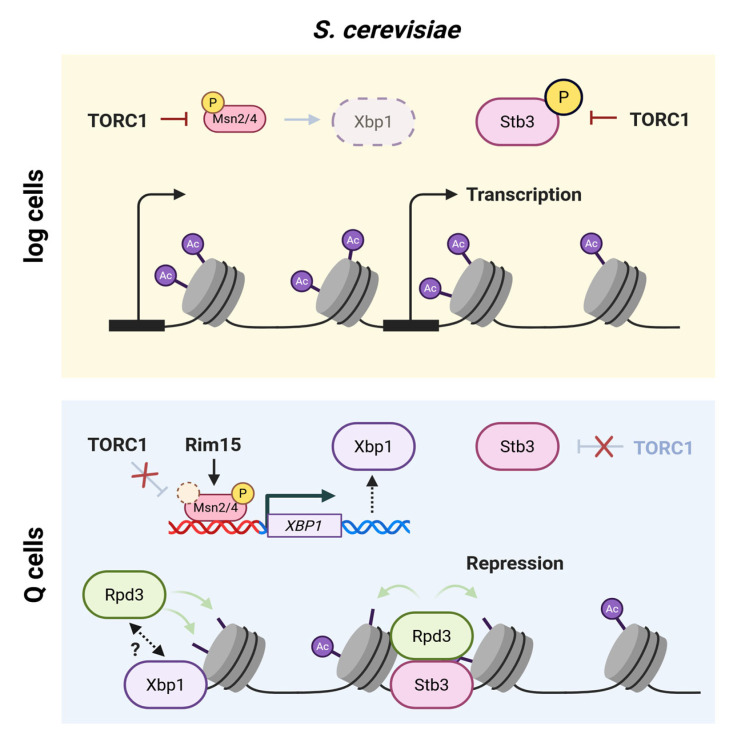
**Mechanisms of transcriptional reprogramming through the concerted actions of repressor proteins.** Histone deacetylation drives quiescence-associated transcriptional repression in *S. cerevisiae.* In log cells (top), high default levels of histone acetylation maintain open promoters and a chromatin structure permissive for transcription, as TORC1 and PKA signaling keep the transcriptional repressors Stb3 phosphorylated and inactive. In Q cells (bottom), TORC1 inactivation activates Stb3, which binds rRNA-processing elements (RRPEs) at ribosome biogenesis genes, and directly recruits the histone deacetylase complex Rpd3L. Concurrently, differential phosphorylation promotes nuclear localization of the stress-regulated transcription factors Msn2 and Msn4, which in return induce *XBP1* expression. Xbp1 associates with Rpd3L in vivo, suggesting direct recruitment to Xbp1-response elements, but the precise molecular mechanism remains unknown (indicated by question mark). Created in BioRender. Kilchert, C. (2026) https://BioRender.com/2hhc1hs (accessed on 17 January 2026).

**Rpd3 is a key mediator of quiescence-specific chromatin remodeling.** Stationary cells lacking the conserved class I histone deacetylase Rpd3 (HDAC1/2 in mammals) display histone acetylation levels and nucleosome density similar to proliferating cells or cells in early quiescence [52]. In log cells, Rpd3 primarily localizes to coding regions and is absent from promoters, whereas in Q cells, Rpd3 redistributes to the promoter regions of approximately 50% of genes [52]. Promoter recruitment is mediated by Stb3 and Xbp1, each of them performing unique, non-redundant roles through targeting distinct sets of genes [52] (Figure 3). Xbp1 has been shown to associate in vivo with the Rpd3L complex, likely via its subunit Ume1 [73], suggesting a direct targeting mechanism for histone deacetylation at specific promoters. Notably, while *rpd3Δ* cells do not show a major growth phenotype in log cells, deletion of *RPD3* or *XBP1* impairs the ability to enter quiescence and reduces chronological lifespan [52]. These findings support a critical role for Rpd3-mediated histone deacetylation in enabling transcriptional and chromatin remodeling as cells enter quiescence, although regulation of non-histone substrates cannot be excluded. Beyond its role in transcriptional repression, bulk histone deacetylation may also contribute to metabolic adaptation by generating free acetate for acetyl-CoA synthesis, thereby supporting energy production and anabolic processes under nutrient-limited conditions [74].

**Restoration of histone acetylation during quiescence exit.** Upon glucose addition, stationary *S. cerevisiae* cells reinitiate transcription in a few minutes and restore histone acetylation before DNA replication resumes, marking their exit from the quiescent state [54,72,75]. Histone H3 and H4 acetylation is mediated by the SAGA and NuA4 histone acetyltransferase complexes, respectively [75]. In particular, TOR signaling promotes NuA4 recruitment to RPG promoters, ensuring that chromatin remodeling and transcriptional activation are tightly coupled to nutrient availability (for a detailed review, see [3]). The rapid re-initiation of transcription is largely dependent on the chromatin remodeling complex RSC that contributes to maintain NDRs upstream of specific genes and help RNAPII to progress into gene bodies upon release [76]. The intergenic accumulation of RNAPII upstream of specific genes including RPGs was also proposed to contribute to their rapid and robust transcriptional activation within minutes of refeeding [57].

**Transcription factor networks in metazoans.** In metazoan cells, quiescence is likewise governed by interconnected transcription factor networks that regulate thousands of genes and integrate nutrient-, stress-, and niche-derived signals to coordinately repress cell cycle genes while activating programs that support stress resistance, metabolic adaptation, and long-term viability. Rather than relying on single master regulators, quiescent mammalian cells deploy combinatorial TF assemblies—including E2F–RB/DREAM complexes, FOXO factors, Notch effectors, and lineage-specific transcription factors—that act through promoter- and enhancer-associated chromatin remodeling to establish reversible cell cycle arrest. As in yeast, these networks are dynamically modulated at multiple levels, including TF localization, stability, post-translational modification, and chromatin accessibility, enabling rapid and synchronous transitions between quiescent and proliferative states. For example, the autophagy-regulating transcription factors TFEB and FOXO3 are retained in the cytoplasm upon phosphorylation by mTORC1 and PI3K-AKT signaling, respectively, but translocate to the nucleus during quiescence following kinase inactivation. For a detailed overview of mammalian transcription factor networks during quiescence, the reader is referred to a recent comprehensive review [77].

### 3.3. Establishment of Silent Chromatin Through Repressive Epigenetic Modifications

In quiescent *S. cerevisiae* cells, nearly half of the genome becomes targeted by Rpd3 [52], driving global histone deacetylation and transcriptional repression. While histone hypoacetylation is a common feature of quiescence across eukaryotes, the mechanisms guiding histone deacetylase recruitment differ substantially. Notably, neither Xbp1 nor Stb3 orthologs are found outside the *Saccharomycetaceae* clade, although functional analogs exist (e.g., the stress-responsive transcription factor Atf1 in *S. pombe*), and rRNA processing element (RRPE)-like sequence motifs at ribosomal synthesis genes are conserved (e.g., the Homo1D and Homo1E motifs in *S. pombe*) [78]. In contrast to budding yeast, fission yeast and many other fungi rely more heavily on repressive histone methylation—a mechanism broadly conserved across metazoans but absent in *S. cerevisiae*—to establish silent chromatin states.

**Mechanisms of constitutive heterochromatin establishment in fission yeast.** In *S. pombe*, silent chromatin is formed through locus-specific recruitment of the histone H3 lysine 9 (H3K9) methyltransferase Clr4, which is targeted through two complementary pathways [79]. Direct recruitment is mediated by sequence-specific DNA-binding factors, such as the heterodimeric transcription factor Atf1/Pcr1. RNA-guided recruitment involves the RNA interference (RNAi) machinery: Dcr1 (dicer) generates small interfering RNAs (siRNAs) that are loaded into Ago1, a core component of the RNA-induced transcriptional silencing (RITS) complex. Ago1-bound siRNAs guide RITS to nascent transcripts through base-paring, while the RNA-directed RNA polymerase Rdp1 amplifies siRNA production to reinforce the signal. Once recruited, Clr4 deposits H3K9me, which is recognized by Heterochromatin Protein 1 (HP1) family members (Swi6, Chp2) via their chromodomains. HP1 oligomerization, mediated by its chromoshadow domain, and additional Clr4 recruitment promote spreading of this histone mark across extended genomic regions. H3K9me is also recognized by RITS via its chromodomain protein Chp1, resulting in additional signal amplification and robust heterochromatin assembly. The resulting heterochromatin domains act as platforms for additional repressive factors, such as the class II HDAC Clr3. In log cells, this machinery establishes repressed chromatin at constitutive heterochromatin domains—pericentromeres, the silent mating type locus, rDNA, telomeres, and subtelomeres—as well as at dispersed facultative heterochromatin islands [79,80].

**Figure 4:**
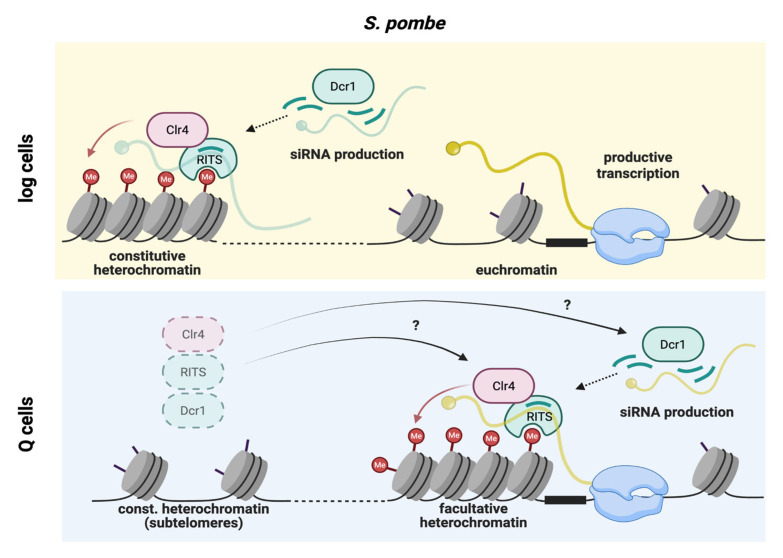
**Repressive histone methylation contributes to transcriptional reprogramming in *S. pombe***. In log cells (top), silencing factors primarily act on constitutive heterochromatin (left). In Q cells, the RNase III enzyme Dicer (Dcr1) redistributes to euchromatic loci to generate small interfering RNAs (siRNAs), supported by RNA-dependent RNA polymerase (Rdp1). These siRNAs guide the RNA-induced transcriptional silencing complex (RITS) to nascent RNA, where it recruits the H3K9 methyltransferase Clr4, depositing repressive marks to form facultative heterochromatin at the silenced loci. RITS is stabilized on heterochromatin through recognition of H3K9me via its chromodomain, thereby reinforcing heterochromatin assembly. For simplicity, assembly of HP1 proteins is not shown. The initial mechanism targeting Dcr1 and RITS to euchromatic loci remains unknown (indicated by question mark), and Clr4 may also be recruited by RNAi-independent mechanisms. Created in BioRender. Braun, S. (2026) https://BioRender.com/t8cro8m (accessed on 17 January 2026).

**Heterochromatin remodeling and function during quiescence.** Emerging evidence indicates that heterochromatin factors play important roles in both quiescence establishment and long-term viability during extended nutrient depletion. Multiple heterochromatin mutants display defects in entering quiescence, and cells lacking Dcr1, Ago1, or Rdp1 show a progressive decline in viability following nitrogen starvation [17,63,81]. Reduced survival of Q cells is also reported for mutants of the Clr6 HDAC complex—the *S. pombe* homolog of *S. cerevisiae* Rpd3L [17]—and for *clr4Δ* cells, although the severity varies across studies [63,81]. During quiescence entry, the heterochromatin landscape is extensively remodeled. Facultative heterochromatin is acquired at approximately 200 euchromatic loci across the genome, marked by de novo H3K9me deposition and accompanied by quiescence-specific siRNAs bound by Ago1 [63] (Figure 4). Many genes downregulated within 24 h of nitrogen starvation—including transcripts with functions in reproduction, carbohydrate metabolism, and transport—require Clr4 for silencing [63]. Consistent with the need to shut down protein biogenesis, the rDNA locus shows increased H3K9me levels and accounts for half of all siRNA production during quiescence, whereas other heterochromatin regions remain largely unchanged [63,81]. An exception are the subtelomeric regions that show a transient decrease in H3K9me levels, concomitant with the upregulation of several subtelomeric genes [63,82]. Consistently, among all surveyed histone methylation marks, both activating and repressive, H3K9me3 exhibits the most pronounced changes [62], underscoring the extent of heterochromatin remodeling.

**Dcr1 functions beyond RNAi.** While these findings support the notion of a key role for RNAi- and Clr4-dependent heterochromatin during early quiescence, genome-wide H3K9me remodeling at later stages, when nearly 97% of the genome is downregulated [64], remains less well explored. Notably, Dcr1 performs an additional, RNAi-independent function at the rDNA locus that is linked to the release of stalled RNAPI and is essential for long-term quiescence survival [81]. Cells lacking Dcr1 accumulate both RNAPI and H3K9me at rDNA repeats at substantially higher levels than in WT during quiescence. Two non-mutually exclusive mechanisms may explain this H3K9me accumulation: First, stalled RNAPI may directly recruit Clr4, in analogy to the recruitment of G9a by RNAPI in mammalian cells [83]. Second, elevated H3K9me levels at rDNA repeats may arise through an indirect mechanism in *dcr1Δ* cells. Specifically, this increase may result from the redistribution of silencing factors following their release from genomic regions that strictly depend on RNAi. This phenomenon has been documented in log cells, where loss of RNAi at one locus leads to enhanced heterochromatin formation at other genomic sites [84,85,86]. Interestingly, the viability defects of *dcr1∆* can be rescued by mutations that impair heterochromatin formation (*clr4Δ*, *swi6Δ*) or by defects in RNAPI recruitment or stability. However, while *clr4Δ* suppresses the viability defects of *dcr1Δ*, it does not prevent RNAPI accumulation. This is in contrast to mutations that impair RNAPI recruitment and stability, which reduce both its accumulation and H3K9me levels in *dcr1*∆ cells [81]. This suggests that RNAPI retention alone is insufficient to account for the viability defect. Rather, although heterochromatin normally accumulates at rDNA during quiescence, excessive H3K9me levels—such as those associated with heterochromatin redistribution or RNAPI retention—are deleterious for survival and therefore require tight regulation. Notably, increased RNAPI association and rRNA accumulation are also observed in cells lacking the coilin homolog Mug174, which associates with Cajal bodies and is implicated in RNA splicing and small ribonucleoprotein biogenesis (see Section 5.1). However, although the absence of Mug174 also leads to viability defects during quiescence, its role is genetically independent of Dcr1 and Clr4, and these defects cannot be suppressed by additional deletion of *clr4.* Instead, this phenotype is more likely directly related to the function of Mug174 in splicing, consistent with the observation that splicing mutants also display quiescence defects [87].

**Diverse repressive histone methylation patterns in metazoans.** Similar regulatory principles, including transcriptional repression and histone hypoacetylation, operate in higher eukaryotes during quiescence, yet the accumulation and function of repressive histone marks vary substantially across cell types and physiological contexts. As in fission yeast, H3K9 methylation in metazoans is closely associated with constitutive heterochromatin at pericentromeric repeats and other repetitive elements. However, H3K9 methylation exhibits no uniform pattern during quiescence: several types of Q cells, such as hair follicle stem cells and adult fibroblasts, show reduced H3K9me2/3, whereas others display moderate increases or little detectable change [72]. A similar heterogenous behavior is observed for H3K27me3. In some Q cell types, including fibroblasts, B lymphocytes, and hair follicle stem cells, H3K27me3 levels decrease together with reduced polycomb repressive complex 2 (PRC2) depositing this mark, whereas MuSCs maintain low promoter H3K27me3 in quiescence but gain this mark upon activation [72,88]. These divergent patterns likely reflect differential use of PRC2 paralogs, with EZH1 prevailing in Q cells and EZH2 associated with proliferative states [72,89]. For a detailed overview of polycomb repressive complex function in adult stem cell quiescence, the reader is referred to a dedicated review [90].

In contrast, H40K20me3 displays a more consistent association with quiescence. H4K20me3 is a repressive mark found in metazoans at pericentromeric heterochromatin and other repetitive elements (including transposable elements and subtelomeric regions) but is absent in *S. cerevisiae* and *S. pombe*. During quiescence, H4K20me3 levels robustly increase across constitutive heterochromatin and can extend into facultative domains, contributing to the establishment of a deeply repressed state [72,91]. Primary human fibroblasts accumulate global levels of H4K20me2 and H4K20me3 during quiescence in association with enhanced chromatin compaction [92], and similar increases occur in serum-deprived mouse NIH 3T3 fibroblasts, where H4K20me3 is enriched at rDNA and transposable elements [93]. Elevated H4K20me3 is likewise observed in MuSCs, whereas loss of this mark induces proliferation and depletion of the MuSC pool [91]. Consistent with a broader role for H4K20me3 in deeply arrested cellular states, drug-tolerant persister cancer cells exhibit marked enrichment of H4K20me3 at promoters of stress-responsive genes, reinforcing transcriptional repression and supporting their low-activity, immune-evading phenotype [94].

**Dynamic remodeling of epigenetic landscapes during mammalian embryonic diapause.** Pluripotent cells in the early embryo, along with embryonic stem cells (ESCs), sustain highly adaptable transcriptional programs that maintain their undifferentiated state through a balance of global permissiveness and localized, polycomb-mediated repression. Entry into quiescence during mouse embryonic diapause triggers a rapid, genome-wide decrease in transcriptional output [6,95], mirroring the suppression of other energy-intensive anabolic processes such as translation. The overall decrease in cellular activity during this state is accompanied by a loss of transcription-associated histone marks, such as H4K16ac and H3K9ac and increased accumulation of heterochromatin at the nuclear lamina in pluripotent epiblast cells [6,96,97,98]. DNA methylation, which is almost completely erased in the proliferative epiblast, likewise globally increases in the diapaused epiblast [99]. Despite this repressive epigenetic landscape that significantly diverges from the canonical state of proliferative epiblast cells, the diapaused epiblast retains the transcriptional signatures and functionality of naïve pluripotency [6,49]. Although many mechanisms that ensure fidelity of cell identity remain to be discovered, several signaling pathways have been shown to protect the epiblast from premature differentiation or cell death during diapause, including Wnt, LIF/Stat, Nodal/Smad, and Yap/Taz pathways [95,100,101]. In addition, TET DNA demethylases, in cooperation with the transcription factor TFE3, have been shown to specifically target cis-regulatory elements and maintain them in a lowly methylated state despite an overall increase in global DNA methylation [99]. Taken together, multiple regulatory layers from cellular signaling to targeted epigenetic maintenance ensure transcriptional fidelity in the face of increased genomic repression in dormant states.

### 3.4. Persistence and Redistribution of Active Histone Methylation Marks

In contrast to the drastic reduction in histone acetylation in *S. cerevisiae*, methylation marks associated with active transcription (H3K4me3, H36me3, and H3K79me3) largely persist when cells enter quiescence [2,53,54]. Genome-wide profiles of these marks correlate well between Q and log cells, yet they differ at specific loci. In particular, all three methylation marks are strongly increased at stress-induced genes early in quiescence, coinciding with a sharp rise in transcription; in contrast, growth-related genes that are transcriptionally repressed exhibit a 50% decrease in H3K4me3 levels, without a comparable reduction of H3K36me3 or H3K79me3 [53]. As cells exit quiescence, histone methylation shifts from stress-related genes to growth genes, yet this redistribution exhibits slower kinetics than the rapid acetylation bursts and occurs only after DNA replication has resumed [54].

Notably, the histone methyltransferases responsible for H3K4me and H3K36me—Set1 and Set2, respectively—are both downregulated in Q cells, suggesting that these histone marks are deposited during logarithmic growth or early in quiescence [53]. Histone demethylases remain expressed in Q cells but may be less active, and decreased replication-dependent and transcription-coupled nucleosome turnover may further contribute to the persistence of these marks [53]. Thus, rather than serving as direct indicators of transcriptional activity, these histone methylation marks may help maintain a chromatin environment that remains permissive to transcription [53,54].

Additional evidence from *S. pombe* further supports the view that H3K4me3 becomes selectively redistributed rather than globally erased during quiescence. Although H3K4me3 is broadly retained, its promoter-proximal peak is markedly diminished in Q cells, accompanied by extensive locus-specific remodeling: after 24 h of nitrogen starvation, hundreds of genes show multi-fold increases or decreases in H3K4me3, even though most promoters lose the sharp TSS-centered peak characteristic of log cells [62]. A subset of quiescence-induced genes retains or gains H3K4me3 together with elevated levels of elongating RNAPII, particularly genes encoding metabolic enzymes, membrane transporters, and noncoding RNAs. Notably, H3K4me3 becomes enriched at ‘core quiescence’ genes, with several of them residing in subtelomeric regions. Their transcriptional induction partially depends on Set1, consistent with genetic evidence that Set1C/COMPASS components are required for efficient quiescence entry [17,62,64]. A comparable pattern is observed in quiescent adult stem cells of higher eukaryotes. However, unlike embryonic stem cells, adult stem cells display limited bivalent H3K4me3/H3K27me3 marks but retain widespread H3K4me3 at promoters, thereby maintaining a transcriptionally poised state that enables rapid activation upon exit from quiescence [38,72]. Together, these findings indicate that, despite global reduction of promoter-centered H3K4me3, the targeted retention and redeployment of this mark contribute to establishing a transcriptionally competent yet selectively reprogrammed chromatin landscape in Q cells.

**H3K79me3: a molecular timer of quiescence duration?** H3K79 methylation is a multifaceted chromatin mark with roles in diverse cellular processes including meiosis and G2/M progression [102]. In *S. cerevisiae*, H3K79me3 remains detectable or even increases in Q cells, while levels of H3K79me1 and H3K79me2 are strongly reduced, suggesting their conversion to the fully methylated state [53]. Intriguingly, this conversion is not observed in NQ cells, which are unable to reenter the cell cycle after prolonged starvation when nutrients are restored, implying a regulatory mechanism involved in maintenance of stable quiescence that remains unidentified [2,53]. Notably, H3K79 methylation is absent in *S. pombe*, indicating this potential timing mechanism is not universally conserved.

### 3.5. Epigenetic Reprogramming of Metabolic Genes Through Altered Histone Dynamics and Nucleosome Remodeling

During quiescence establishment, numerous genes become transiently or persistently upregulated in *S. pombe* [17,55,63,64,82]. Many of these genes are located in subtelomeric regions and encode vacuolar transmembrane transporters essential for recycling amino acids and hexoses during the metabolic shift of Q cells. Multiple genetic studies converge on the conclusion that this transcriptional activation depends on regulated histone exchange and nucleosome remodeling, processes that remain active even when DNA replication—and thus replication-coupled histone turnover—is halted.

**Ino80C mediates the removal of the histone variant H2A.Z.** A major component of this regulatory network is the Ino80C chromatin-remodeling complex (Ino80C), which repositions nucleosomes and catalyzes the removal of the histone variant H2A.Z (Pht1 in *S. pombe*). Loss of Ino80C subunits, such as Iec1 (ino eighty complex subunit 1), compromises survival during quiescence and induces premature, global transcriptional downregulation [17,64]. Accordingly, genes normally upregulated at quiescence onset in WT cells fail to be activated in *iec1Δ* and other Ino80C mutants. In line with this, whereas H2A.Z is normally depleted from subtelomeric regions during quiescence entry, it remains aberrantly enriched in *iec1*∆, particularly at boundary elements that contain multiple long terminal repeat (LTR) sequences [64]. These findings imply that Ino80-mediated eviction of H2A.Z promotes the formation of a transcriptionally competent subtelomeric environment, facilitating activation of metabolic genes required for the establishment of the quiescent state.

This dependency is further modulated by inositol polyphosphate signaling: the inositol kinase Asp1, which influences Ino80C activity, is likewise required for survival under prolonged quiescence [64,103], underscoring the integration of metabolic cues with chromatin remodeling at this transition. Conversely, the H2A.Z-specific histone chaperone Swr1C is dispensable for viability during quiescence, indicating that H2A.Z removal, rather than its deposition, is critical at this stage [17]. Nonetheless, loss of H2A.Z itself also causes reduced viability [64], suggesting a distinct, Ino80C-independent function for this variant.

**Paf1C and histone turnover contribute to epigenetic reprogramming.** The Paf1 complex (Paf1C) promotes histone exchange during vegetative growth and counteracts the accumulation of H3K9 methylation at constitutive and facultative heterochromatin [104]. Q cells lacking Leo1 or other Paf1C components exhibit substantial viability defects [82], highlighting the importance of ongoing, DNA replication-independent histone replacement. In WT cells, heterochromatic H3K9me2/3 levels transiently decline during quiescence entry, particularly at subtelomeric regions; this decrease is attenuated in *leo1Δ*, resulting in elevated H3K9me and reduced expression of transporter genes required for metabolic adaptation [82]. Conversely, other gene clusters, including those involved in DNA repair and chromatin organization, become aberrantly upregulated, reflecting broader disruptions to transcriptional control.

Genetic and pharmacological evidence further positions Leo1 downstream of the TORC2–Gad8^Akt^ pathway: in fission yeast, TORC2 is required to maintain heterochromatin integrity at subtelomeric regions, where it stabilizes repressive chromatin states and limits inappropriate transcription. Through this activity, TORC2 helps preserve epigenetic stability by restraining transcriptional permissiveness [105]. Conversely, TORC2 inhibition by torin induces transcriptional changes that depend on Leo1, while Gad8^Akt^ protein abundance transiently decreases during quiescence entry [106]. These relationships suggest that TORC2 signaling modulates Paf1C-dependent histone turnover to enable epigenetic reprogramming of metabolic pathways, particularly during the early phase of quiescence establishment [5,82].

Together, these findings illustrate that histone variant dynamics and replication-independent histone turnover are central determinants of gene regulation during quiescence. Ino80C-dependent H2A.Z eviction and Paf1C-mediated histone replacement act in concert to remodel subtelomeric chromatin, permit activation of key metabolic genes, and maintain viability during long-term quiescence.

## 4. Post-Transcriptional Mechanisms Shape Quiescence-Specific Gene Expression

### 4.1. Intron Retention Regulates Protein Biogenesis Genes

Transcriptional adaptations during quiescence are accompanied by significant changes in co-transcriptional RNA processing, particularly splicing [107]. Although *S. cerevisiae* has largely lost splicing capacity during evolution, retaining introns in only 5% of its genes, introns are nevertheless strikingly preserved in ~73% of ribosomal protein genes (RPGs). During amino acid starvation, but not in response to other stress conditions, splicing of RPG mRNAs is selectively repressed, and strains lacking these introns exhibit reduced fitness during prolonged starvation [108,109,110]. This suggests that regulated intron retention within RPGs functions as a post-transcriptional mechanism to attenuate ribosome biogenesis, thereby limiting energy expenditure as cells enter quiescence. Notably, a subset of excised introns accumulates as a stable, linear form in saturated *S. cerevisiae* cultures or after TORC1 inhibition. These stable introns associate with spliceosomes and may themselves contribute to reduced splicing activity under starvation conditions [111]. A subset of stress-responsive genes escapes splicing inhibition and is even more efficiently spliced under starvation conditions, correlating with their induction. These transcripts selectively recruit auxiliary splicing factors, including the U1-associated proteins Nam8 and Mud1, to promote 5′ splice site recognition and intron removal [109].

**Figure 5:**
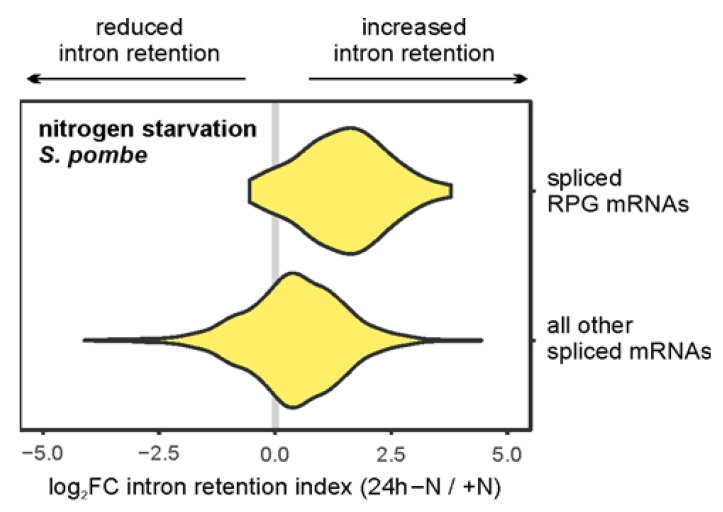
**Increased intron retention regulates ribosome synthesis genes during quiescence.** Comparison of intron retention rates in *S. pombe* during log phase (+N) and after 24 h of nitrogen starvation (−N) for mRNAs encoding ribosomal proteins and all other spliced mRNAs. Intron retention index based on published RNA-seq data (E-MTAB-1154; Ref. [55]) calculated as the ratio of intronic reads over exonic reads for each transcript.

In *S. pombe*, where splicing is more prevalent (~46% of genes contain introns), transcriptome profiling after 24 h of nitrogen starvation reveals a global increase in intron retention, with RPGs again being particularly affected [55] (Figure 5). This conservation across evolutionarily divergent yeast species indicates that splicing repression of RPGs represents a fundamental regulatory mechanism in quiescence establishment. Consistently, widespread intron retention is also a hallmark of mammalian quiescent adult stem cells, with enrichment among transcripts encoding proteins involved in translation and splicing [112,113]; diapaused embryos likewise exhibit reduced splicing activity [48]. Functionally, intron retention appears to be essential for maintaining quiescence: activation of MuSCs requires quiescence exit, which can be blocked by removing the chromatin-associated factor Dek that promotes intron removal [112].

Beyond population-level changes, RPG splicing can also vary among individual cells within populations, creating phenotypic heterogeneity with distinct consequences for adaptation to stress. In *S. cerevisiae*, the RPG Rps22B exhibits a bimodal splicing pattern, with different subpopulations displaying different survival outcomes during starvation: cells with intron retention (low levels of Rps22B) show enhanced survival during prolonged starvation, while cells with efficient intron removal (high levels of Rps22B) resume growth more quickly after transient starvation [114]. Stochastic cell-to-cell variation may represent a bet-hedging strategy that exploits phenotypic diversity to maximize fitness in unpredictable environments. Remarkably, regulated splicing of an intron in the 5′ untranslated region (5′-UTR) is conserved in the homologous *S. pombe* gene, *rps2202*, where it activates a post-transcriptional decay mechanism that is specifically inactivated during nitrogen starvation in homothallic yeast [115,116].

In mammalian cells, mTORC1 signaling regulates alternative splicing patterns more broadly. Serine/arginine-rich (SR) proteins, which play a central role in splice site selection, are controlled by phosphorylation via the SR protein kinase SRPK2. mTORC1-S6K signaling promotes nuclear translocation of SRPK2, thereby linking mTORC1 activity to splicing regulation [117].

### 4.2. 3′-UTR Lengthening Expands the Repertoire of Post-Transcriptional Regulation

Quiescence is characterized by widespread shifts in polyadenylation site (PAS) selection. While proliferating cells preferentially use proximal PAS and thereby generate transcripts with short 3′-untranslated regions (3′-UTRs), non-proliferating cells (including both differentiated and Q cells) more commonly use distal PAS, resulting in global 3′-UTR lengthening [118,119]. This shift toward distal PAS usage appears to be a conserved feature of Q cells across eukaryotes, and has been documented in *S. pombe* and *S. cerevisiae* grown under nutrient-poor conditions [120], in *S. pombe* after 24 h and 7 d of nitrogen starvation [121], or upon rapamycin treatment [122].

**Molecular mechanisms driving 3′-UTR extension.** Kinetic coupling between transcription and 3′-end processing renders PAS selection highly sensitive to both the transcription elongation rate and the availability of the 3′-end processing machinery. Slow transcription and abundant 3′-end processing factors favour proximal PAS usage and 3′-UTR shortening, whereas faster elongation or reduced processing factor availability shifts selection towards distal sites [123,124]. Accordingly, chromatin changes that modulate elongation kinetics, together with the reduced expression of RNA cleavage and polyadenylation factors in Q cells, are expected to shift the equilibrium towards distal PAS usage and 3′-UTR lengthening [55,63,113]. Emerging evidence indicates that nutrient-sensing pathways help coordinate these shifts in PAS usage. In *S. cerevisiae*, rapamycin-induced alternative PAS selection requires the methyltransferases Set1 and Set2, linking TOR inhibition to transcription-dependent histone modifications (see above; [122]). In mammalian cells, hyperactivation of mTORC1 also drives 3′-UTR shortening, selectively enhancing translation of genes involved in ubiquitin-mediated proteolysis and facilitating targeted degradation of cell-cycle regulators without altering global mRNA abundance [125].

**Regulatory consequences of 3′-UTR extension.** Extended 3′-UTRs typically harbor additional binding sites for RNA-binding proteins (RBPs) and microRNAs, thereby increasing the potential for post-transcriptional control. In differentiated cells, this expanded regulatory landscape generally attenuates gene expression by enhancing opportunities for transcript destabilization or translational repression. Whether this paradigm also applies to Q cells, however, remains unclear. RNA stability measurements in a dermal fibroblast model of quiescence revealed stabilization, rather than destabilization, of transcripts with extended 3′-UTRs [113], suggesting that Q cells may exploit 3′-UTR lengthening to achieve regulatory outcomes distinct from those observed in differentiated tissues. This observation aligns with recent analyses in *S. cerevisiae* showing that the impact of 3′-UTR isoforms on mRNA half-life is highly condition-dependent, with transcripts becoming progressively more stable when cells divide more slowly [126].

**Suppression by microRNAs.** In metazoans, microRNAs (miRNAs) provide an important layer of post-transcriptional control acting at the level of 3′-UTRs. These small RNAs function exclusively as repressors, either suppressing translation or inducing RNA cleavage in a sequence-specific manner after incorporation into RNA-induced silencing complexes (RISC) [127]. Interestingly, hypoxia-inducible factor 1α (HIF-1 α), a transcription factor involved in the generation and maintenance of quiescent cancer stem cells developing in hypoxic niches [128], appears to additionally dampen miRNA-dependent regulation. HIF-1α sequesters the miRNA processing component Dgcr8 and prevents its incorporation into microprocessors, thereby limiting miRNA production in the cancer stem cell niche [129]. However, evidence from multiple other mammalian quiescence models suggests that miRNAs actively regulate quiescent states. In MuSCs, for example, the quiescence-specific miRNA-489 maintains quiescence by repressing Dek along with other targets [130]. Similarly, entry into embryonic diapause involves extensive reprogramming of miRNA expression [48,131,132]. The upregulation of diapause-associated miRNAs is partly controlled by the nutrient-responsive transcription factors Tfe3/TfeB, which act downstream of mTOR signaling. Upon mTOR inhibition, these factors partially translocate to the nucleus and promote miRNA expression [48]. While miRNAs are dispensable for normal blastocyst development, they are critical for initiating diapause, evidenced by the inability of embryos and embryonic stem cells lacking Dgcr8 to enter dormancy [48,133]. One key miRNA, let-7—originally identified as a regulator of developmental timing in *C. elegans*—is upregulated during diapause and plays a role in modulating embryo implantation [131,134]. Let-7 inhibits *Myc*, mTOR signaling, and polyamine biosynthesis, thereby promoting a diapause-like state [134]. Given that embryonic diapause is predominantly regulated by maternal cues, it has been proposed that endometrial extracellular vesicles—key mediators of communication between the uterus and the embryo—deliver let-7 miRNA to the blastocyst to help establish the dormant state [134].

### 4.3. Regulatory Non-Coding RNAs Facilitate Rewiring of Gene Expression

**Figure 6:**
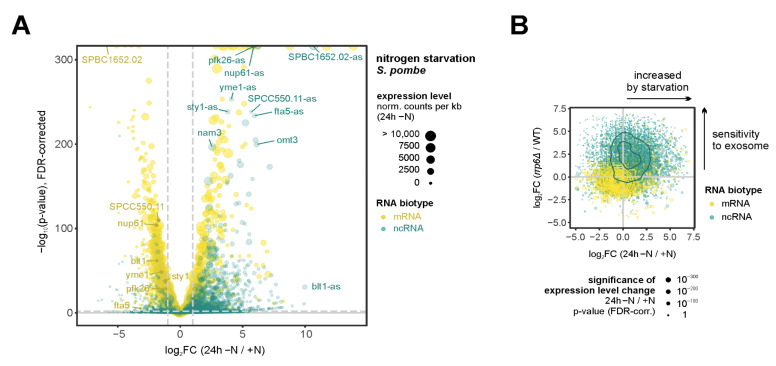
**Accumulation of non-coding RNAs is a conserved feature of quiescent cells.** (**A**). Differential gene expression analysis in *S. pombe* comparing RNA levels during log phase (+N) and after 24 h of nitrogen starvation (−N) for mRNAs (yellow) and ncRNAs (turquoise) (DESeq2). *p*-values adjusted for multiple testing with the Benjamini-Hochberg procedure to control the false discovery rate (FDR). Size of circles indicates absolute expression levels in nitrogen-starved cells (read counts per kilobase). RNA-seq data from E-MTAB-1154 (Ref. [55]; *n* = 2). (**B**)**.** Comparison of RNA expression level changes in nitrogen-starved cells and cells that lack the exonuclease Rrp6, a catalytic subunit of nuclear exosome for mRNAs (yellow) and ncRNAs (turquoise). Size of circles indicates statistical significance of the expression level change (FDR-corrected *p*-values). Contours of the 2d density estimates for both RNA biotypes are shown in yellow and turquoise, respectively. RNA-seq data from E-MTAB-1154 (Ref. [55]; *n* = 2) and GSE148799 (Ref. [140]; *n* = 3).

A striking and conserved feature across diverse quiescence models is the widespread upregulation of non-coding RNAs (ncRNAs). Transcriptome-wide analyses in human somatic quiescence have identified numerous upregulated long non-coding RNAs (lncRNAs), and elevated ncRNA expression is similarly observed in both budding and fission yeast [55,56] (Figure 6A). This conservation across evolutionarily distant organisms suggests that ncRNA accumulation represents a fundamental feature of Q cells, rather than a species-specific adaptation.

**What drives ncRNA accumulation in quiescence?** Beyond transcriptional changes, reduced RNA decay rates are thought to contribute to the distinct lncRNA profiles observed in Q cells. In proliferating cells, nuclear RNA stability depends largely on susceptibility to degradation by the nuclear exosome, a conserved exonucleolytic complex central to RNA surveillance that is guided to its substrates by a modular system of targeting factors [135]. In Q cells, levels of many exosome substrates are substantially increased (Figure 6B). Three non-mutually exclusive mechanisms have been proposed to explain this phenomenon: (i) reduced exosome expression or activity (in fission yeast, exosome mRNA levels decrease to ~50% [63]); (ii) altered exosome targeting, either through inactivation of specific targeting pathways or activation of quiescence-specific mechanisms (e.g., HIF1α-dependent retargeting of human microprocessor protein Dgcr8 to the exosome [129,136]); or (iii) substrate competition, where retargeting of the exosome to a broader substrate pool, including incompletely processed mRNAs with retained introns or extended 3′-ends (see above), creates kinetic competition that stabilizes ncRNAs [137,138]. By itself, exosome depletion in mouse embryonic stem cells induces nuclear structural changes that resemble senescent cells, including chromatin compaction [139].

**Figure 7:**
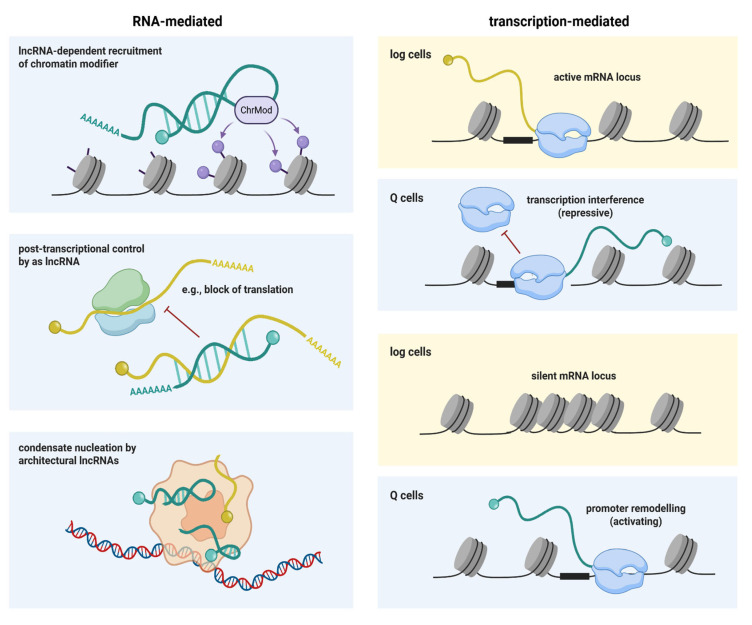
**Multiple mechanisms of lncRNA-dependent regulation operate during quiescence.** lncRNA expression can regulate gene expression through mechanisms mediated by the RNA molecule itself (**left**). These include recruiting epigenetic modifiers to chromatin to alter local histone marks and transcription (**top**); base-pairing with a complementary RNA to alter protein binding and its expression (**middle**); or nucleating biomolecular condensates that control the stability or activity of sequestered mRNAs (**bottom**). Alternatively, the act of transcription can be regulatory independent of the lncRNA product (**right**). This can involve transcriptional interference, where lncRNA synthesis competes with and represses an adjacent gene (**top**), or promoter remodeling, where the passage of RNA polymerase opens an occluded promoter to activate a nearby gene (**bottom**). RNA biotype indicated by colour—mRNAs (yellow) and ncRNAs (turquoise). Created in BioRender. Kilchert, C. (2026) https://BioRender.com/i8yei16 (accessed on 17 January 2026).

Although the regulatory potential of quiescence-associated lncRNAs is clear, only few have been functionally characterized, sometimes in contexts unrelated to quiescence. LncRNAs, or the act of their transcription, can regulate cellular functions through various mechanisms, or a combination of them, including:

**Recruitment of epigenetic modifiers:** Some lncRNAs recruit chromatin-modifying enzymes to specific loci through direct interaction or via RBP intermediaries, establishing epigenetic signatures that regulate quiescence programs (Figure 7). This mechanism is conserved from yeast to mammals. In fission yeast, the lncRNA *prt* recruits H3K9 methyltransferase Clr4 to silence the adjacent *pho1* locus under phosphate-replete conditions but is transcriptionally downregulated when cells are starved for phosphate [141]. This recruitment involves a sequence-specific RBP and a scaffold protein mediating the interaction [80]. In metazoans, similar lncRNA-dependent targeting mechanisms regulate chromatin compaction during quiescence. While the histone H4K20 methyltransferase Suv4-20h2 is typically recruited to constitutive heterochromatin in an HP1-dependent manner, mouse Q cells employ an alternative HP1-independent mechanism. Quiescence-induced lncRNAs produced in cis at ribosomal RNA repeats and other repetitive regions recruit Suv4-20h2, potentially through direct interaction with the enzyme, promoting chromatin compaction at repeat regions [93] (Figure 7).

**ncRNA-mRNA interactions:** Other lncRNAs modulate activity of individual target mRNAs through base-pairing interactions, leading to a range of potential outcomes. The lncRNA NR2F1-AS1, for example, is significantly upregulated in human breast cancer stem-like cells, which are known to give rise to metastatic persister cells in the lungs. NR2F2-AS1 functionally contributes to establishing or maintaining the quiescent state—its knock-down leads to increased proliferation [142]. Mechanistically, NR2F1-AS1 binds an inhibitory region within the 5′-UTR of NR2F1 mRNA, facilitating recruitment of the polypyrimidine tract-binding protein PTBP1 and thereby promoting translation of the transcription factor NR2F1. Similarly, the fission yeast lncRNA *aal1* (ageing-associated lncRNA 1), has been suggested to suppress proliferation by interacting with mRNAs that encode ribosomal proteins, limiting their expression and decreasing the cellular ribosome content [143] (Figure 7).

**Architectural lncRNAs and condensate nucleation.** Many lncRNAs are large, structurally flexible molecules capable of interacting with multiple proteins through various binding sites. Such multivalent interactions can result in the formation of biomolecular condensates with high densities of RBPs that spatially or temporally regulate RNA-dependent processes [144] (Figure 7). The protein components of nuclear condensates often contain intrinsically disordered low-complexity regions that contribute to the network of multivalent interactions. A notable example of such condensates are paraspeckles—mammalian-specific nuclear structures that require a specific RNA scaffold, NEAT1 lncRNA, for their formation—which sequester specific RBPs and RNAs [145]. In *S. pombe*, certain quiescence-associated lncRNAs, such as *omt3* [55] (Figure 6A), form condensates at the non-coding gene locus. During meiosis (when these lncRNAs are also induced), such condensates facilitate the pairing of homologous chromosomes through a tethering mechanism [146]. The proteins involved in these condensates are components of the RNA 3′-end processing machinery that, in log cells, localize to cleavage bodies—structures thought to buffer nucleoplasmic concentrations of 3′-end formation factors and not known to be associated with lncRNAs [147]. Their behavior in Q cells remains uncharacterized.

**Regulation through lncRNA transcription.** The act of lncRNA transcription—rather than the lncRNA product itself—can strongly influence the expression of adjacent genes through distinct and context-dependent mechanisms. In transcriptional interference, passage of RNAPII through a promoter region can displace transcription factors necessary for initiation, effectively suppressing gene expression [148] (Figure 7). This repressive mechanism operates independently of gene orientation and can occur when the lncRNA gene and the regulated gene are oriented in tandem or when they are in an antisense configuration. Silencing of the *S. pombe pho1* locus by *prt* lncRNA also depends on this mechanism [141]. Transcriptional interference appears to be widely deployed during quiescence: in *S. cerevisiae*, even short periods of glucose or phosphate starvation induce distinct sets of antisense lncRNAs (asRNAs) [149,150]. In *S. pombe*, asRNAs are among the most strongly upregulated ncRNAs in Q cells and often accumulate to high levels [55,151]. This elevated expression typically coincides with downregulation of the corresponding sense gene, illustrating the regulatory impact of transcriptional interference (Figure 6A). Widespread changes in antisense transcription have also been observed in human models of senescence, with a subset of asRNAs showing inverse expression patterns relative to their corresponding sense transcript [152]. Moreover, recent work using synthetic antisense loci in mouse cells demonstrates that antisense transcription can establish stable promoter repression that persists for days, suggesting that transcriptional interference may create a form of expression memory [153]. Conversely, lncRNA transcription can facilitate gene activation through promoter remodeling. When RNAPII traverses compacted chromatin, transcription itself disrupts nucleosome organization, rendering previously inaccessible promoters competent for transcription factor binding. At the *S. pombe fbp1* locus, for example, glucose starvation induces cascading lncRNA transcription, which precedes and enables activation of *fbp1* mRNA transcription [154]. Such pioneering lncRNA transcription, which primes chromatin for productive mRNA synthesis, has been observed at other genes responding to nutritional signals, and may represent a widespread mechanism for transcriptional reprogramming during quiescence entry and exit [155].

## 5. Reorganization of Nuclear Structures and Genome Architecture

### 5.1. Biosynthetic Condensates

**Nucleolar condensation as a conserved marker of quiescence.** The biogenesis of core components of the gene expression machinery is spatially organized in specialized nuclear condensates whose morphology reflects cellular biosynthetic activity. Nucleoli serve as hubs for ribosome maturation, while spliceosome and small nucleolar ribonucleoprotein (snoRNP) production occurs in distinct compartments (see below). During quiescence, nucleoli undergo dramatic reorganization, condensing into compact structures as ribosomal RNA (rRNA) synthesis declines with growth arrest. This condensation is a highly conserved hallmark of Q cells, observed across phylogenetically distant organisms and triggered by different cues: in *S. cerevisiae* following DS [156]; in *S. pombe* cells during nitrogen starvation [33,157]; in *C. elegans* under insulin pathway inhibition [158]; and in mouse embryos during diapause [98]. This reorganization is reversible: upon exit from diapause, nucleoli decondense within 12 h, coinciding with restored biosynthetic activity in the reactivating embryos [96,98]. To date, the molecular composition of condensed nucleoli remains largely unexplored. Ultrastructural analysis of quiescent *S. pombe* nuclei (nitrogen-starved for 24 h) revealed an accumulation of multi-mega Dalton molecular complexes in the condensed nucleolus [157], but their nature and functional role remain uncharacterized.

**Cajal bodies coordinate small RNA biogenesis with growth state.** Cajal bodies are nuclear structures essential for the maturation of small nuclear RNAs (snRNAs) and small nucleolar RNAs (snoRNAs) [145]. These RNAs are highly abundant components of the gene expression machinery: snRNAs form the catalytic core of the spliceosome, while snoRNAs guide the chemical modification of rRNA required for ribosome biogenesis. Because their production contributes significantly to the biosynthetic load, snRNA and snoRNA biogenesis is tightly coordinated with nutrient availability. In fission yeast, the Cajal body scaffold protein Mug174 (the orthologue of metazoan coilin) is required for viability of Q cells and their subsequent return to proliferation, indicating that long-term survival depends on Cajal body-mediated coordination of small ribonucleoprotein biogenesis with the cellular growth state [87]. The molecular mechanisms underlying this nutrient-responsive regulation are better characterized in mammalian systems, where TOR signaling plays a central role: the survival of motor neurons (SMN) protein, responsible for assembling sn(o)RNPs from sn(o)RNAs and Sm proteins, is a direct mTOR target. Cajal body localization of SMN—which reflects its activity—is regulated by mTOR-dependent phosphorylation, providing a direct link between nutrient sensing, sn(o)RNP biogenesis, and the cellular capacity of RNA splicing [159]. Intriguingly, not all organisms employ Cajal body-based regulation. *S. cerevisiae* lacks orthologues of coilin or SMN, and the site and mechanism of sn(o)RNP assembly in budding yeast remain unresolved. Assembly may occur diffusely within the nucleus, without discrete compartmentalization, raising the question of which alternative mechanisms coordinate small RNA biogenesis with nutrient availability in this organism.

### 5.2. Changes in Local and Higher-Order Chromatin Structures

Quiescence induces profound reorganization of genome architecture at both the local chromatin level and the genome-wide scale, resulting in dramatic genome compaction and nuclear volume decrease across species [2]. In *S. cerevisiae*, these changes arise through two complementary mechanisms involving histone tail–dependent chromatin fiber compaction and condensin-mediated chromosome looping. Together, these processes generate a deeply compact, insulated, and transcriptionally restrained nuclear architecture tailored to the low-energy state of Q cells.

**Local chromatin remodeling through chromatin fiber compaction.** In budding yeast Q cells, neighboring nucleosomes engage in interactions dependent on the basic patch of the H4 histone tail, a process facilitated by global hypoacetylation of H4 [160]. This mode of fiber compaction is thought to broadly dampen transcription, largely independent of higher-order domain architecture. While mechanistically distinct, similar principles apply in human quiescent fibroblasts, where chromatin compaction is associated with increased H4K20me2/3 levels [92]. In *S. cerevisiae*, an additional contributor might be the histone H1 homolog Hho1. Hho1 chromatin occupancy is inversely correlated with gene expression, and it binds more strongly to chromatin in stationary-phase cells, with its loss leading to chromatin decompaction [161]. Notably, this decompaction does not result in widespread transcriptional derepression, indicating that chromatin compaction alone is not sufficient to enforce gene silencing [2,161].

**Global chromosome reorganization by condensin.** At a global scale, the quiescent genome is reorganized into a highly ordered structure defined by long-range interactions and condensin-mediated looping. Hi-C analyses of *S. cerevisiae* Q cells reveal a shift toward a more compact nuclear organization characterized by increased intrachromosomal contacts, reduced centromere clustering, and enhanced telomere–telomere interactions [156,162,163,164]. At the mesoscale, chromatin is organized into chromosomal interaction domains (CIDs), small genomic units encompassing 1–4 genes (0.5–8 kb) that engage in frequent local interactions and resemble metazoan topologically associating domains [165]. Superimposed on this architecture are larger chromatin interaction domains (L-CIDs) spanning 10–60 kb, which are present in both log and Q cell and whose boundaries remain largely unchanged. However, during quiescence, L-CIDs become more compact and topologically isolated, accompanied by enhanced loop formation [164]. Together, these global rearrangements indicate a transition to a condensed yet orderly chromatin state that is distinct from both log and stationary-phase cells.

A critical determinant in this process is the condensin complex, a ring-shaped protein complex that organizes higher-order chromosome structure and mediates sister chromatid cohesion [162,164]. During quiescence entry, condensin redistributes from its canonical locations at rDNA and centromeres to the 5′ ends of hundreds of stress-induced genes, aligning with L-CID boundaries. This relocalization is partially dependent on the transcription factor Msn2, which is involved in the activation of many stress-induced genes during the DS (see above). How Msn2 contributes to condensin binding is not well understood, but a role of transcription itself at Msn2-responsive genes has been discussed [59]. Loss of condensin in G0 leads to decompaction of L-CIDs, a reduction in loop formation, increased inter-domain contacts, and transcriptional derepression of nearby genes [164], underscoring its role as an architectural organizer that enforces domain insulation during quiescence. How L-CID boundaries are established, which occurs cohesin-independently in log cells, remains unresolved.

### 5.3. Telomere Reorganization

**Telomere hypercluster formation in *S. cerevisiae.*** In budding yeast, telomere organization undergoes extensive remodeling during metabolic transitions. Following the diauxic shift, telomere foci become fewer and brighter while remaining associated with the nuclear periphery. Upon complete carbon source exhaustion and entry into stationary phase, telomeres undergo a more pronounced reorganization in long-lived Q cells, coalescing into a large hypercluster positioned at the nuclear center [156]. This configuration is reversible: telomere hyperclusters are rapidly dismantled within 15 to 30 min upon return to growth, in a transcription-independent manner [57]. Importantly, telomere hypercluster formation correlates with survival upon quiescence exit and is specific to Q cells, as telomere organization remains unchanged in NQ cells [156].

The extent of telomere hyperclustering varies markedly between strain backgrounds. W303 strains exhibit robust hyperclustering, with telomere foci showing a moderate increase in intensity and remaining at the nuclear periphery in most cells of a stationary-phase culture [163,166]. Although early work proposed that elevated Sir3 levels might account for this difference [163], this model has been excluded: moderate Sir3 overexpression (2–3.5-fold) in BY fails to rescue the telomere hyperclustering defect, and Sir3 protein levels remain stable across metabolic transitions in both backgrounds [156,166]. Instead, reduced hyperclustering in BY strains likely reflects respiration defects [167], consistent with respiration being a key requirement for telomere hypercluster formation [156]. Notably, Sir3 post-translational modifications, including SUMOylation and phosphorylation, are dispensable for telomere hyperclustering in Q cells [166].

Recent physical modeling and genetic analyses identified telomere anchoring as a key negative regulator of telomere hyperclustering [166]. The inner nuclear membrane protein Esc1 acts as a major telomere anchor upon glucose exhaustion; in *esc1∆* cells, telomere hyperclusters form prematurely, indicating that Esc1-mediated anchoring counteracts telomere clustering. Under glucose-replete conditions, however, hyperclusters do not form in *esc1∆* cells, implying the existence of an Esc1-independent anchoring mechanism. This secondary anchoring pathway is regulated by glucose signaling, as glucose starvation or inactivation of the Ras/PKA pathway triggers hyperclustering in *esc1∆* cells [166]. In wild-type Q cells, exhaustion of all carbon sources coincides with hypercluster formation, consistent with inactivation of Esc1 anchoring, whereas in NQ cells Esc1 remains active and prevents hypercluster formation [166].

Mechanistically, the Esc1-dependent anchoring pathway relies on phosphorylation of Esc1 at serine 1450, which enables interaction with the HBRCT domain of Sir4 [168,169]. Mutation of this residue phenocopies Esc1 loss, suggesting that reversible Esc1 phosphorylation controls telomere anchoring during metabolic transitions, although the upstream signaling cascade remains unknown [166].

Together, these findings indicate that budding yeast has evolved a fine-tuned mechanism to regulate telomere positioning in response to nutrient availability. While telomere hyperclustering promotes cell survival following exit from quiescence independently of transcriptional silencing, its precise protective function remains unknown [156]. Hyperclustering may enhance genome stability by protecting chromosome ends from degradation, end-to-end fusions, and aberrant recombination events. Alternatively, telomere cluster dynamics may influence the transcriptional regulation of subtelomeric genes, which are enriched in stress-response and metabolic genes, thereby coordinating cellular physiology with environmental conditions [170].

**Telomere hypercluster formation in *S. pombe.*** As in budding yeast, entry into quiescence in *S. pombe* is accompanied by a pronounced spatial reorganization of telomeres, most notably their collapse into a single hypercluster. In *S. pombe* log cells, telomeres form two to three nuclear envelope (NE)-associated clusters during interphase, an organization that persists through DNA replication [171,172,173]. The shelterin component Rap1 directly contributes to telomere tethering at the NE through interaction with the NE-associated Bqt3-Bqt4 complex [172,174]. NE localization is reinforced by subtelomeric anchoring mediated by the inner nuclear membrane LEM-domain proteins Lem2 and Man1, which associate with distinct subtelomeric regions [85,175,176]. Simultaneous loss of Bqt4 and the chromatin remodeler Fft3, which interacts with Man1, results in relocalization of telomeres toward the nuclear interior [175].

Upon quiescence entry, telomeres collapse into a single cluster. In contrast to *S. cerevisiae*, where telomere hyperclusters form in the center of the nucleus, fission yeast telomeres remain tightly anchored to the NE during nitrogen starvation, in a manner dependent on Rap1 and Bqt4. Nuclear polarity is maintained, with telomeres localizing opposite the spindle-pole body (SPB), where centromeres are anchored, thereby preserving the Rabl configuration [177]. Importantly, loss of Bqt4 does not alter the number of telomeric foci, indicating that NE tethering and telomere hyperclustering represent mechanistically distinct steps [177]. Disruption of NE attachment, either by loss of Bqt4 or telomere erosion, leads to increased telomeric transcription and predisposes cells to subtelomeric rearrangements via a quiescence-specific repair mechanism termed STEEx (STE1-expansion) [177,178]. Cells harboring rearranged telomeres fail to efficiently exit quiescence [178]. Telomere clustering has also been reported for quiescent mouse and human lymphocytes [179], supporting the notion that telomere repositioning during quiescence may represent a conserved strategy to preserve genome stability in non-proliferating cells [180].

### 5.4. Dynamic Relocalization of Stress-Induced Gene Clusters

In *S. pombe*, stress-response genes are frequently organized into physical clusters along chromosomes, consistent with coordinated regulation shaped by chromatin architecture and nuclear organization [181]. Several clusters that are rapidly induced upon nitrogen starvation are located near subtelomeres and associate with the nuclear periphery in proliferating cells, but relocalize to the nuclear interior upon starvation, coincident with their activation [182]. Because the nuclear periphery is generally considered a repressive environment enriched in silencing factors [183,184], these observations raised the question of whether gene repositioning is a cause or a consequence of transcriptional activation. Notably, similar behavior is observed for the *urg1–3* gene cluster, which is located in a euchromatic region rather than at subtelomeres. Despite this genomic context, the cluster associates with the nuclear periphery during growth and relocates to the nuclear interior upon nitrogen starvation, providing a useful model to study regulated gene repositioning independently of constitutive subtelomeric silencing.

Early experiments using the broad transcriptional inhibitor 1,10-phenanthroline suggested that cluster relocalization depends on transcription [182] However, given the pleiotropic effects of this compound, transcription-independent effects could not be excluded. More targeted perturbations support an alternative model, in which relocalization precedes transcription. The transcription factor Toe1 is required for the induction of genes within the *urg* cluster, yet deletion of *toe1* abolishes transcriptional activation without preventing relocalization of the cluster to the interior in starved cells, arguing that repositioning occurs upstream, or independent, from transcription (S.B. and Vishnu N. Suma Sreechakram, unpublished data).

Consistent with its euchromatic context, perinuclear tethering of the *urg* cluster does not require the inner nuclear membrane protein Lem2, a key factor in heterochromatin silencing and subtelomeric anchoring ([85] S.B. and Vishnu N. Suma Sreechakram, unpublished data). Instead, a putative NE-associated repressor complex, Ntu1-Ntu2, binds a promoter within this cluster, and loss of this complex phenocopies starvation-induced relocalization, implicating Ntu1-Ntu2 in anchoring the cluster at the nuclear periphery [185]. How nitrogen starvation signals trigger release of this tether, and whether additional NE proteins contribute to positioning of other stress-induced clusters, remain open questions.

### 5.5. Impact of Nuclear Reorganization on Genome Functions

As described above, nuclear reorganization is associated with genome stability, gene regulation, and the long-term viability of Q cells. However, establishing causal relationships between nuclear structures and functions remains challenging. Some structural changes arise as a consequence of functional transitions, while simultaneously conferring adaptive advantages. For example, chromatin and nucleolus compaction result, at least in part, from genome-wide deacetylation and transcriptional shutdown; however, these changes may also contribute to long-term genome stability. Likewise, nuclear microtubules become extensively reorganized upon carbon exhaustion in budding yeast, forming a single array of approximately 30 microtubules that emanate from the spindle pole body and traverse the nucleus, resulting in the displacement of the nucleolus [186]. This microtubule bundle is surprisingly stable and disappears upon quiescence exit. Mutants perturbing microtubule organization display defects in long-term survival and upon quiescence exit. While a role as a structural gatekeeper controlling quiescent exit has been proposed, the precise physiological function of this rearrangement remains unresolved [187]. Microtubule remodeling during quiescence has also been reported for *S. pombe*, plants and mammalian cells; however, these alterations predominantly involve cytoplasmic microtubules and the primary cilium [187,188]. Although genetic perturbations affecting nuclear reorganization have clear functional consequences, indirect effects are difficult to exclude and defects in quiescence entry, maintenance, or exit are often hard to disentangle. Inducible protein degradation strategies applied specifically in Q cells will therefore be essential to minimize pleiotropic effects and dissect the molecular requirements of these distinct phases.

## 6. Quiescence: Quo Vadis?

Work across yeast and metazoan models has firmly established quiescence as an actively regulated program rather than a passive cessation of proliferation. Nevertheless, major challenges remain in resolving its underlying mechanisms. A central open question concerns the physiological function of the diverse nuclear rearrangements observed in Q cells: Do these changes act as causal drivers of quiescence or instead arise as downstream consequences of altered transcription, metabolism, and chromatin state? This question has not yet been resolved for the repositioning of stress-responsive gene clusters, telomere hyperclustering, global chromosome compaction, remodeling of nuclear bodies, and extensive reprogramming of RNA metabolism. For many of these phenomena, their functional contribution to quiescence entry, transcriptional adaptation, and long-term survival remain poorly defined.

A second major frontier is cellular heterogeneity, which complicates both mechanistic interpretation and conceptual definitions of quiescence. Quiescent populations typically comprise mixtures of Q and NQ cells, and even highly enriched Q populations likely span a continuum of states. This raises foundational questions including whether cells commit to quiescence long before proliferation ceases, whether previous experience of quiescence alters the speed of re-entry (is there a memory of quiescence?), when quiescence is firmly established, and whether there are differences in the “depth” of the quiescent state (see article in this special issue by Marek et al., Ref. [20]). Addressing these issues necessitates a shift from viewing quiescence as a single fixed endpoint toward a framework of layered or graded quiescence states, characterized by time-dependent changes in signaling output, chromatin dynamics, and gene expression, potentially relying on molecular timers that encode a physical memory of quiescence duration (see review in this special issue by Laporte and Sagot, Ref. [7]). Progress in this area will rely on single-cell and longitudinal approaches capable of resolving transition trajectories during entry, maintenance, and exit.

A third emerging theme centers on RNA-mediated nuclear regulation, including non-coding RNAs and post-transcriptional control. Q cells typically globally reduce transcriptional output while selectively accumulating subsets of long non-coding RNAs, raising the possibility that components of the “dark transcriptome” exert regulatory functions that become apparent only in non-proliferative states. Beyond intron retention, future studies should systematically examine isoform usage, RNA stability control, nuclear-cytoplasmatic partitioning, and RNA-protein condensate dynamics as mechanisms that preserve readiness for renewed proliferation while suppressing energetically costly biogenesis.

Finally, quiescence-specific nuclear dynamics connect to development and disease. Quiescence underpins adult stem cell maintenance and immune homeostasis, while quiescence-like programs contribute to both therapy resistance of cancer persister cells and to drug tolerance of chronic or recurrent fungal infections. Quiescence research offers potential new therapies for these important medical challenges. A comparative perspective remains essential: how conserved are nuclear quiescence programs across species and triggers, and which features represent cell type-specific adaptations rather than universal molecular pathways. Can this heterogeneity be selectively targeted for therapeutic purposes? Addressing these questions will require systematic cross-system analyses to identify condition- and disease-specific nuclear implementations of quiescence.

## 7. Conclusions

Quiescence is accompanied by extensive nuclear reorganization, encompassing the chromatin architecture, chromosome positioning, nuclear body dynamics, and RNA metabolism. While these changes are now well documented across eukaryotes, we are only beginning to understand their mechanistic basis and functional consequences. TOR signaling emerges as a central coordinator linking nutrient availability to nuclear architecture, though many mechanistic details remain unresolved. Understanding these mechanisms has direct clinical relevance: therapy-resistant persister cells in both cancers and fungal infections exploit quiescence programs to evade treatment, and their distinctive nuclear features may represent targetable therapeutic vulnerabilities. Moving forward, resolving the broader interplay between nuclear structure and function during quiescence and understanding how nuclear dynamics contribute to quiescence entry, maintenance, and exit represents a major frontier for the field.

## Figures and Tables

**Table 1:** Glossary of key terms and abbreviations.

Abbreviation	Definition	Explanation	Species Context
G0	G0 phase	Reversible cell-cycle arrest in which cells stop dividing but retain the ability to re-enter proliferation.	Conserved
Q cells	Quiescent cells	Cells in a stable, non-proliferative state with reduced biosynthesis and extensive nuclear remodeling; also referred to as reversible G0 cells.	Conserved
NQ cells	Non-quiescent cells	Cells that fail to achieve deep quiescence and exist in an unstable intermediate state, transitioning more rapidly than Q cells into irreversible senescence during prolonged nutrient deprivation	Functionally conserved
log cells	Proliferating cells	Exponentially growing cells dividing at the maximal rate that conditions allow; often referred to as “logarithmic” because cell number plotted against time on a semi-logarithmic scale yields a straight line.	Conserved
Diapause	Embryonic diapause	Reversible developmental arrest of the mammalian embryo, typically at the blastocyst stage.	Metazoans (mammals)
TOR	Target of Rapamycin	Nutrient-sensing kinase pathway coordinating growth, metabolism, and quiescence.	Yeast
mTOR	Mechanistic Target of Rapamycin	Metazoan TOR pathway integrating nutrient and growth-factor signaling to regulate dormancy and growth.	Metazoans
TORC1	TOR complex 1	TOR-containing complex promoting ribosome biogenesis and translation; inhibited during quiescence.	Conserved
TORC2	TOR complex 2	TOR-containing complex regulating stress responses and survival pathways.	Conserved
PKA	Protein kinase A	cAMP-dependent kinase promoting growth-associated transcription and antagonizing quiescence.	Conserved
DS	Diauxic shift	Metabolic transition in which yeast cells switch from fermentative growth on glucose to respiratory metabolism of alternative carbon sources after glucose depletion.	*S. cerevisiae*
MuSC	Muscle stem cell	Quiescent adult stem cells involved in muscle maintenance and repair; also known as satellite cells	Metazoans
RNAPII	RNA polymerase II	Enzyme transcribing mRNAs and many non-coding RNAs; redistributes during quiescence.	Conserved
RNAPI	RNA polymerase I	Enzyme transcribing ribosomal RNA genes at the rDNA locus; tightly linked to growth state.	Conserved
RiBi	Ribosome biogenesis	Coordinate transcriptional program encoding ribosomal proteins, rDNA processing factors, and ribosome assembly components.	*S. cerevisiae* (conserved growth regulon in eukaryotes)
RRPE	rRNA processing element	Cis-regulatory promoter motif enriched in ribosome biogenesis genes that mediates coordinated transcriptional repression via binding of Stb3. Related elements (Homo1D and Homo1E motifs) also exist in *S. pombe*.	*S. cerevisiae*
RP(G)	Ribosomal protein(s) (gene(s))	Structural and functional components of ribosomes; their expression is tightly repressed during quiescence.	Conserved
Stb3	Sin3-binding protein 3	Nutrient-regulated transcriptional repressor of RiBi genes that recruit Rpd3 to promote chromatin compaction and biosynthetic shutdown	*S. cerevisiae*
Xbp1	XhoI site-binding protein 1	Stress-induced transcriptional repressor that binds promoter regions of growth-associated genes	*S. cerevisiae*
HDAC	Histone deacetylase	Enzymes removing acetyl groups from histones, promoting chromatin compaction and repression.	Conserved
Rpd3	Reduced potassium dependency 3	Class I HDAC broadly retargeted to promoters during budding yeast quiescence.	*S. cerevisiae*
Set1C/COMPASS	Set1 complex	H3K4 methyltransferase complex depositing H3K4me3 at promoters.	Conserved
SAGA	Spt-Ada-Gcn5 acetyltransferase complex	Transcriptional coactivator complex with histone acetyltransferase and deubiquitylation activities that promotes transcription initiation and stress- and growth-related genes.	Conserved
H3K4me3	H3 lysine 4 trimethylation	Active promoter mark selectively retained or redistributed during quiescence.	Conserved
H3K36me3	H3 lysine 36 trimethylation	Histone modification associated with transcription elongation and gene body marking, largely maintained during quiescence (in yeast).	Conserved
Ino80C	Ino80 chromatin remodeling complex	ATP-dependent remodeler evicting H2A.Z and regulating transcription during quiescence.	Conserved
H2A.Z	H2A.Z histone variant	Histone variant modulating promoter responsiveness and chromatin boundaries.	Conserved
Hho1	Histone H1 homolog	Linker histone contributing to chromatin compaction without enforcing full silencing.	*S. cerevisiae*
Paf1C	Polymerase-associated factor 1 complex	Transcription elongation complex that associates with RNAPII and regulates histone modifications linked to active transcription	Conserved
Clr3/Clr6	Cryptic loci regulators 3/6	HDACs contributing to heterochromatin formation and quiescence maintenance.	*S. pombe*
Clr4	Clr4 methyltransferase	Histone H3K9 methyltransferase/Suvar 39 h homolog, heterochromatin initiation and spreading.	*S. pombe*
H3K9me	H3 lysine 9 methylation	Repressive histone mark defining constitutive and facultative heterochromatin.	Conserved (absent in *S. cerevisiae*)
H3K27me3	H3 lysine 27 trimethylation	Polycomb-associated repressive mark controlling facultative heterochromatin repression.	Metazoans
H4K20me3	H4 lysine 20 trimethylation	Deeply repressive mark associated with chromatin compaction and long-term arrest.	Metazoans
PRC2	Polycomb repressive complex 2	Complex depositing H3K27me3 to mediate stable transcriptional repression.	Metazoans
EZH1	Enhancer of zeste homolog 1	PRC2 catalytic subunit prevalent in non-proliferative and Q cells.	Metazoans
EZH2	Enhancer of zeste homolog 2	PRC2 catalytic subunit associated with proliferation and activation.	Metazoans
HP1	Heterochromatin protein 1	Chromodomain protein binding H3K9me and promoting heterochromatin spreading.	Conserved (functional analogs in yeast)
RNAi	RNA interference	Small RNA–guided silencing pathway coupling transcripts to heterochromatin formation.	*S. pombe*, metazoans
Dcr1	Dicer	RNase III enzyme producing siRNAs; also performs RNAi-independent roles in quiescence survival.	*S. pombe*
Ago1	Argonaute 1	Small-RNA effector protein targeting transcripts and chromatin.	*S. pombe*, metazoans
RITS	RNA-induced transcriptional silencing complex	RNAi effector complex linking siRNAs to chromatin-based repression.	*S. pombe*
lncRNA	Long non-coding RNA	>200-nt RNAs regulating chromatin, transcription, and nuclear organization.	Conserved
asRNA	Antisense RNA	Non-coding RNA transcribed opposite a gene, often mediating transcriptional interference.	Conserved
PAS	Polyadenylation site	RNA cleavage site determining 3′-UTR length and post-transcriptional regulation.	Conserved
3-UTR	3′-untranslated region	mRNA region controlling stability, localization, and translation efficiency.	Conserved
CID	Chromosomal interaction domain	Local self-interacting chromatin unit detected by Hi-C.	Yeast (conceptually conserved)
L-CID	Large chromosomal interaction domain	Larger chromatin domains that become more insulated during quiescence.	*S. cerevisiae*
Condensin	Condensin complex	SMC complex organizing higher-order chromosome structure via loop formation.	Conserved

**Table 2:** The nuclear hallmarks and molecular effectors of the quiescent state in unicellular and multicellular organisms described in this review. Superscript 1: yeast (*S. cerevisiae* or *S. pombe*); superscript 2: metazoans or mammalian cells.

Cellular Process	Hallmarks/Phenotype	Nuclear Mechanism/Factors
Cell cycle	Stable arrest with ability to re-enter proliferation	TOR ^1^/mTOR ^2^ inhibition, PKA ^1^ signaling, Myc ^2^ downregulation
Growth and metabolism	Reduced protein synthesis and metabolic activity	Repression of ribosomal RNA and protein biogenesis ^1,2^, translational control ^1,2^, intron retention ^1,2^
Autophagy and survival	Increased autophagy and stress resistance	TOR ^1,2^ inactivation, nutrient sensing pathways ^1,2^
Transcriptional regulation	Global transcriptional repression with selective gene activation	RNAPII redistribution ^1,2^, transcriptional repressors (Xbp1 ^1^, Stb3 ^1^), chromatin remodeling ^1,2^
Chromatin state	Increased chromatin compaction and heterochromatin formation	HDACs (Rpd3 ^1^, Clr3 ^1^, Clr6 ^1^), histone methyltransferases (Clr4 ^1^, PRC2 ^2^)
Histone modifications	Global hypoacetylation, accumulation of repressive methylation marks	Histone deacetylation ^1,2^, H3K9 ^1,2^/H3K27 ^2^/H4K20 ^2^ methylation pathways
Genome organization	Nuclear and 3D genome reorganization	Condensin ^1,2^, heterochromatin dynamics ^1,2^, telomere clustering ^1^, stress-induced gene cluster relocalization ^1^
RNA abundance	Strong reduction in total mRNA levels	Reduced RNAP II initiation and elongation ^1,2^, RNA decay modulation ^1,2^
RNA processing	Widespread intron retention	Splicing repression ^1,2^, spliceosome retention ^1^, TOR signaling ^1^
Post-transcriptional regulation	3′-UTR lengthening, altered RNA stability	Polyadenylation site selection bias ^1,2^, reduced cleavage ^1^
Transcript storage	Accumulation of RNAs in condensates	RNA-Protein phase separation ^1,2^
Non-coding RNA levels	Increased lncRNAs and other ncRNAs	Reduced nuclear exosome activity ^1,2^, lncRNA-mediated recruitment of chromatin modifiers ^2^
Stress and metabolic genes	Relative enrichment of stress-response and nutrient scavenging transcripts	Ino80C ^1^, Paf1C ^1^, histone turnover ^1^, replication-independent chromatin remodeling ^1^
Re-proliferation capacity	Transcriptional reactivation upon nutrient readdition	Histone acetyltransferases (SAGA ^1^, NuA4 ^1^), TOR reactivation ^1^

## Data Availability

No new data were created or analyzed in this study.
